# Generation of Alveolar Epithelial Spheroids via Isolated Progenitor Cells from Human Pluripotent Stem Cells

**DOI:** 10.1016/j.stemcr.2014.07.005

**Published:** 2014-08-21

**Authors:** Shimpei Gotoh, Isao Ito, Tadao Nagasaki, Yuki Yamamoto, Satoshi Konishi, Yohei Korogi, Hisako Matsumoto, Shigeo Muro, Toyohiro Hirai, Michinori Funato, Shin-Ichi Mae, Taro Toyoda, Aiko Sato-Otsubo, Seishi Ogawa, Kenji Osafune, Michiaki Mishima

**Affiliations:** 1 Department of Respiratory Medicine, Graduate School of Medicine, Kyoto University, Kyoto 606-8507, Japan; 2 Institute for Integrated Cell-Material Sciences (iCeMS), Kyoto University, Kyoto 606-8507, Japan; 3 Center for iPS Cell Research and Application (CiRA), Kyoto University, Kyoto 606-8507, Japan; 4 Department of Pathology and Tumor Biology, Graduate School of Medicine, Kyoto University, Kyoto 606-8507, Japan

## Abstract

No methods for isolating induced alveolar epithelial progenitor cells (AEPCs) from human embryonic stem cells (hESCs) and induced pluripotent stem cells (hiPSCs) have been reported. Based on a study of the stepwise induction of alveolar epithelial cells (AECs), we identified carboxypeptidase M (CPM) as a surface marker of NKX2-1^+^ “ventralized” anterior foregut endoderm cells (VAFECs) in vitro and in fetal human and murine lungs. Using *SFTPC-GFP* reporter hPSCs and a 3D coculture system with fetal human lung fibroblasts, we showed that CPM^+^ cells isolated from VAFECs differentiate into AECs, demonstrating that CPM is a marker of AEPCs. Moreover, 3D coculture differentiation of CPM^+^ cells formed spheroids with lamellar-body-like structures and an increased expression of surfactant proteins compared with 2D differentiation. Methods to induce and isolate AEPCs using CPM and consequently generate alveolar epithelial spheroids would aid human pulmonary disease modeling and regenerative medicine.

## Highlights

• Carboxypeptidase M (CPM) is a marker of alveolar epithelial progenitor cells
• CPM is useful for isolating “ventralized” anterior foregut endoderm cells (VAFECs)
• 3D coculture of CPM^+^ VAFECs enables alveolar differentiation
• *SFTPC-GFP* knockin reporter hPSCs help to detect and isolate SFTPC^+^ cells

## Graphical Abstract

**Figure:**
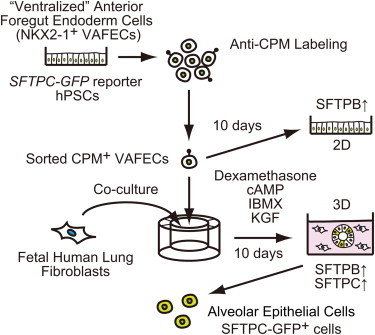

## Teaser

No methods for isolating induced alveolar epithelial progenitor cells (AEPCs) from human pluripotent stem cells (hPSCs) have been reported. Gotoh, Ito, and colleagues developed *SFTPC-GFP* knockin reporter hPSCs and demonstrated that carboxypeptidase M is useful for isolating AEPCs and generating alveolar epithelial cells in a 3D coculture system.

## Introduction

Type II alveolar epithelial cells (AECs) are a major cellular component of the distal lung epithelium, where they secrete pulmonary surfactant and generate type I AECs that cover most of the surface area of the alveoli (Whitsett et al., 2010; Rock and Hogan, 2011). The stepwise differentiation of human pluripotent stem cells (hPSCs), including human embryonic stem cells (hESCs) and induced pluripotent stem cells (hiPSCs), into lung epithelial cells would help to elucidate the etiologies of human lung diseases and create novel treatments, and has been reported in both proximal airway cells (Mou et al., 2012; Wong et al., 2012; Firth et al., 2014) and distal lung epithelial cells (Green et al., 2011; Ghaedi et al., 2013; Huang et al., 2014). Currently, however, there are no surface markers that can be used to purify human NKX2-1^+^ “ventralized” anterior foregut endoderm cells (VAFECs) as alveolar epithelial progenitor cells (AEPCs), although NKX2-1 is an early marker of lung and thyroid development (Kimura et al., 1996). Here, we report the efficacy of carboxypeptidase M (CPM) as a surface marker of AEPCs for generating type II AECs.

## Results

### Identification of CPM as a Marker of NKX2-1^+^ VAFECs

**Figure 1:**
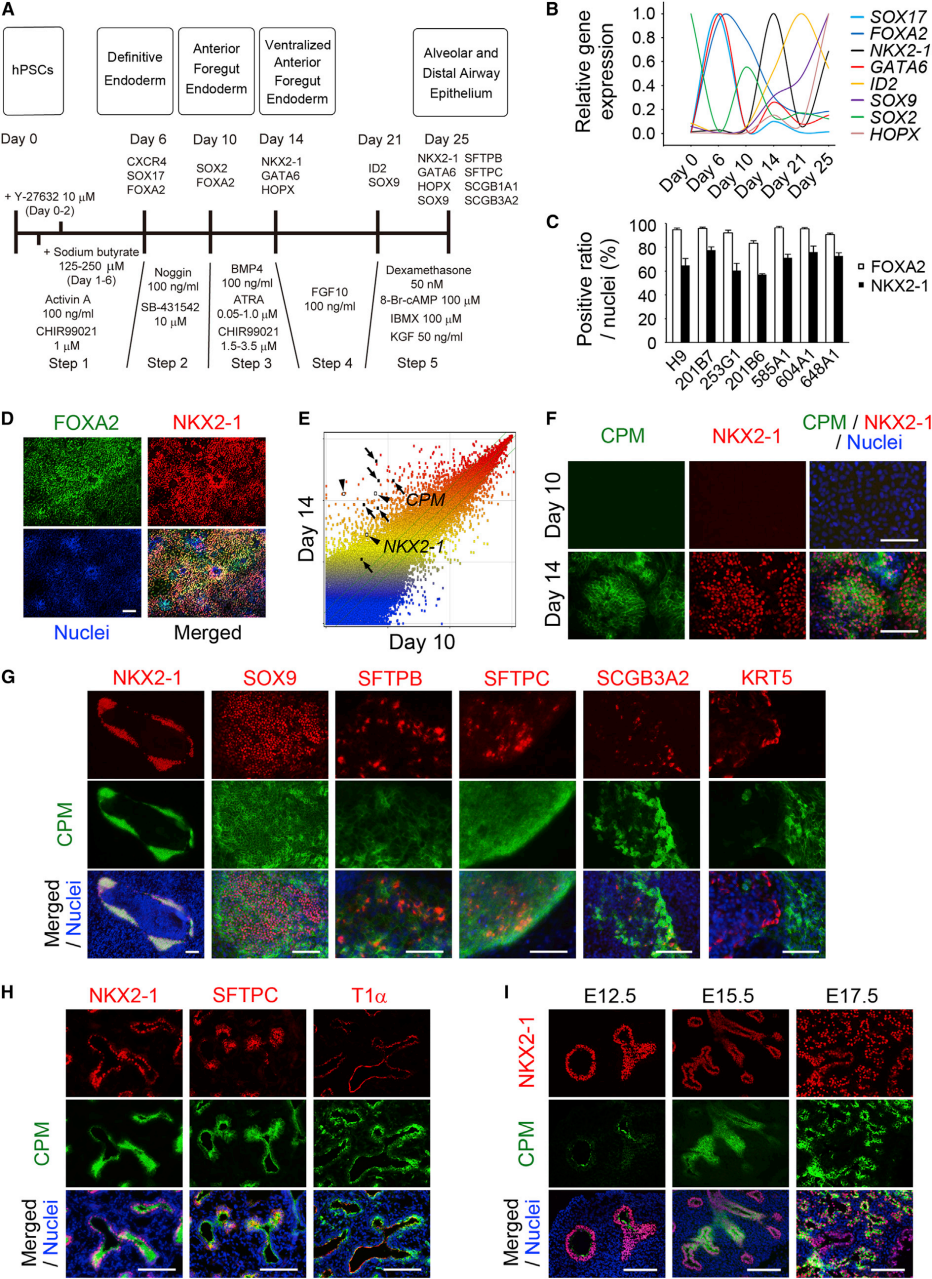
Identification of CPM as a Candidate Marker of NKX2-1^+^ VAFECs (A) Stepwise differentiation to AECs from hPSCs. (B) Gene-expression levels of transcription factors from day 0 to day 25 (n = 3). Each value was normalized to the level of *β-ACTIN*. The relative expression level was scored with the maximum value set to 1.0. (C) Induction efficiency of VAFECs analyzed by scoring the number of FOXA2^+^ and NKX2-1^+^ cells relative to the total number of nuclei in an average of ten randomly selected images (n = 3). (D) FOXA2^+^NKX2-1^+^ VAFECs derived from 201B7 hiPSCs. (E) Scatterplots comparing the global gene-expression profiles of AFECs (day 10) and VAFECs (day 14). *CPM* (arrows) and *NKX2-1* (arrowheads) are noted. The lines beside the diagonal line indicate a 2-fold cutoff change between the AFECs and VAFECs. (F) Simultaneous increases of CPM and NKX2-1 detected by IF staining of AFECs (day 10) and VAFECs (day 14). (G) CPM detected in NKX2-1^+^, SOX9^+^, SFTPB^+^, SFTPC^+^, and SCGB3A2^+^ cells, but not in KRT5^+^ cells, on day 25. (H) CPM detected in NKX2-1^+^ lung epithelial cells in fetal human lung. (I) CPM in E12.5, E15.5, and E17.5 murine lungs. Error bars show SEM. Scale bars, 100 μm. See also Figure S1 and Tables S1 and S2.

We hypothesized that identifying a surface marker for NKX2-1^+^ VAFECs would be helpful for isolating a homogeneous population of AEPCs without establishing *NKX2-1* reporter cell lines. We constructed a stepwise protocol to induce hPSCs to AECs (Figure 1A). On day 0, previously established hPSCs were seeded (Thomson et al., 1998; Takahashi et al., 2007; Nakagawa et al., 2008; Okita et al., 2013) following single-cell enzymatic dissociation (Kajiwara et al., 2012), resulting in definitive endodermal cells (DECs) at an efficiency of ≥80% (Figure S1A available online). In step 2, the DECs were differentiated to anterior foregut endodermal cells (AFECs) (Green et al., 2011) at an efficiency of ≥88% (Figure S1B). In step 3, the concentrations of all-*trans* retinoic acid, CHIR99021, and BMP4 were optimized for seven hPSC lines for differentiation into NKX2-1^+^FOXA2^+^ cells, attaining an efficiency of 57.0%–77.5% (Figures 1C and 1D; Supplemental Experimental Procedures). In step 4, cells were cultured in medium containing FGF10 for 7 days. In step 5, the cells were differentiated in medium containing dexamethasone, 8-Br-cAMP, 3-isobutyl-1-methylxanthine, and KGF (Gonzales et al., 2002; Longmire et al., 2012). We confirmed induction of AECs by detecting *SFTPB* and *SFTPC* using RT-PCR and double staining SFTPC and SFTPB with NKX2-1 (Figures S1C and S1D). Transcription factors were analyzed by quantitative RT-PCR (qRT-PCR; Figure 1B). *SOX17*, *FOXA2*, *GATA6*, and *SOX2* were compatibly changed on day 6 and day 10 as previously described (Green et al., 2011). On day 14, *NKX2-1*, *GATA6*, *ID2*, *SOX9*, and *HOPX* levels simultaneously increased. Interestingly, *NKX2-1*, *GATA6*, and *HOPX* levels decreased on day 21 and then increased again on day 25. The levels of other organ lineage markers were found to be limited from day 0 to day 25 (Figure S1E).

In order to identify candidate markers of VAFECs, we performed a microarray analysis to compare the global gene-expression patterns of AFECs (day 10) and VAFECs (day 14) in 201B7 hiPSCs. *CPM* and *NKX2-1* were remarkably upregulated on day 14 (Figures 1E and S1F). In immunofluorescence (IF) staining, CPM and NKX2-1 increased from day 10 to day 14 (Figure 1F), whereas EPCAM and FOXA2 did not appear to change (Figure S1G). Although CPM was reported to be a marker of type I AECs (Nagae et al., 1993), only *CPM* drastically increased on day 14 in a similar pattern to *NKX2-1*, in contrast to other markers of type I AECs (*AQP5* and *CAV1*) (Figure S1H). On day 25, the various airway markers, including distal lung transcription factors (SOX9 and NKX2-1), type II AEC markers (SFTPB and SFTPC), and a club cell marker (SCGB3A2), were expressed in the CPM^+^ cells. KRT5, a marker of basal cells, was not expressed in the CPM^+^ cells (Figure 1G).

In fetal human lung at 18.5 weeks of gestation, SFTPC and T1α were expressed separately (Figure S1I), while CPM was expressed in NKX2-1^+^, SFTPC^+^, and T1α^+^ cells (Figure 1H), thus indicating that CPM is expressed in both type I and II AECs in the fetus. The sequential expression of CPM was confirmed in NKX2-1^+^ cells of fetal murine lungs at embryonic day 12.5 (E12.5), E15.5, and E17.5 (Figure 1I). For thyroid lineage cells, which differentiated from NKX2-1^+^ VAFECs, CPM was found to be negative in both NKX2-1^+^ cells and PAX8^+^ epithelial cells, but weakly positive in PECAM^+^ endothelial cells in the adult human samples (Figure S1J). In E17.5 fetal and adult murine thyroids, CPM was also negative in NKX2-1^+^ and PAX8^+^ cells (Figure S1K), suggesting that CPM is a lung-lineage marker of VAFECs.

### Isolation of NKX2-1^+^ VAFECs Using Anti-CPM^+^ Antibody

**Figure 2:**
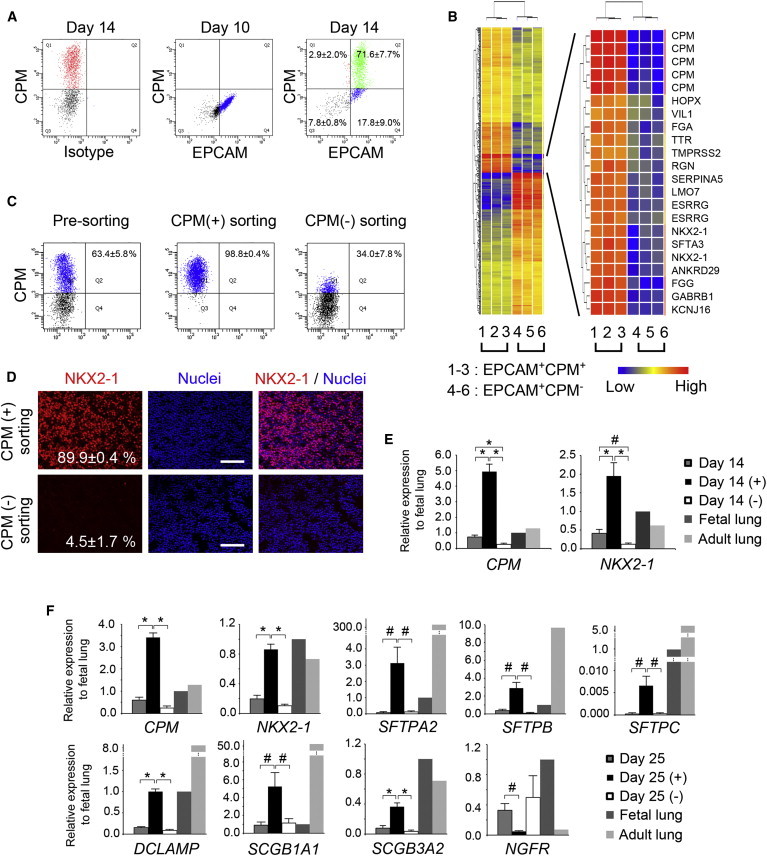
Isolation of CPM^+^ VAFECs Using Anti-CPM Antibody (A) Flow cytometry of VAFECs. EPCAM^+^CPM^+^ (Q2) and EPCAM^+^CPM^−^ cells (Q4) were isolated on day 14 (n = 3). (B) Hierarchical clustering heatmaps of 336 genes with differences of >2-fold (FDR-adjusted p < 0.05) comparing EPCAM^+^CPM^+^ cells with EPCAM^+^CPM^−^ cells. The cluster of genes increased as the greatest fold change was magnified. (C) Flow cytometry of MACS-sorted CPM^+^ and CPM^−^ cells from VAFECs (n = 3). (D) NKX2-1^+^ cells in FACS-sorted CPM^+^ and CPM^−^ cells derived from VAFECs analyzed by scoring the number of NKX2-1^+^ cells relative to the total number of nuclei in an average of five randomly selected images (n = 3). (E) Levels of *CPM* and *NKX2-1* on day 14 before and after MACS-based purification of CPM^+^ cells on qRT-PCR (n = 5). (F) Levels of AEC and club-cell markers and *NGFR*, a proximal airway stem cell marker, on day 25 before and after MACS-based purification of CPM^+^ cells (n = 5). The gene-expression level observed in the fetal lungs was set at one. Values are presented as the mean ± SEM. Error bars show SEM. #p < 0.05, ^∗^p < 0.01. Scale bars, 100 μm. See also Figure S2 and Tables S1 and S2.

As CPM is a membrane-bound surface protein, we performed flow cytometry with anti-EPCAM and anti-CPM antibodies after dissociating VAFECs on day 14 (Figures 2A and S2A). We then sorted EPCAM^+^CPM^+^ and EPCAM^+^CPM^−^ cells and examined the global gene-expression patterns of these two populations using microarrays. We screened 560 probes with a false discovery rate (FDR)-adjusted p value of <0.05 among 54,675 probes. Gene clustering was performed in 336 probes that differed between the EPCAM^+^CPM^+^ and EPCAM^+^CPM^−^ cells with a fold change (FC) cutoff value of 2.0 (Figures 2B and S2B). Of the clustered genes with the highest expression in the EPCAM^+^CPM^+^ cells, *CPM* ranked among the top five probes with a log FC of >6, as expected. Importantly, the log FCs of two probes for *NKX2-1* were 4.89 and 4.82, respectively. *FOXA1*, *FOXA2*, *HOPX*, and *GATA6* were also included in the list of upregulated genes with log FCs of 3.79, 3.06, 3.61, and 3.29, respectively. Next we sorted the CPM^+^ cells using a magnet-activated cell sorting (MACS) system to increase the yield, as almost all of the CPM^+^ cells were EPCAM^+^ cells (96.7% ± 2.1% of CPM^+^ cells; Figure 2A). After MACS-based sorting, the proportion of CPM^+^ cells in three populations (presorting, positive selection, and negative selection) was 63.4% ± 5.8%, 98.8% ± 0.4%, and 34.0% ± 7.8%, respectively, by flow cytometry (Figure 2C). We then evaluated the proportion of positive NKX2-1^+^ cells among the MACS-sorted CPM^+^ and CPM^−^ cells using IF staining (93.0% ± 1.0% versus 29.0% ± 1.0%; Figure S2C) and flow cytometry (92.3% ± 0.7% versus 22.2% ± 2.3%; Figure S2D). Because a portion of the CPM^+^ cells appeared to be sorted according to MACS-based CPM negative selection, we investigated the average proportion of NKX2-1^+^ cells among the fluorescence-activated cell sorting (FACS)-sorted CPM^+^ and CPM^−^ cells using IF staining (89.9% ± 0.4% versus 4.5% ± 1.7%; Figure 2D). Following CPM-based purification on day 14, *CPM* increased significantly from 0.74-fold ± 0.12-fold to 4.94-fold ± 0.51-fold of that observed in the fetal human lung (n = 5), while *NKX2-1* increased from 0.41-fold ± 0.10-fold to 1.95-fold ± 0.36-fold (n = 5) on qRT-PCR (Figure 2E). We then applied this method to purify AECs on day 25. *CPM, NKX2-1*, *SFTPA2*, *SFTPB*, *SFTPC*, *DCLAMP*, *SCGB1A1*, and *SCGB3A2* were significantly increased in the CPM^+^ cells (n = 5); however, the level of *SFTPC* was extremely low compared with that observed in the fetal lung. *NGFR*, a marker of proximal airway basal stem cells (Rock et al., 2009), was significantly decreased in the CPM^+^ cells (n = 5; Figure 2F).

### Generation of *SFTPC-GFP* Knockin Reporter hPSCs

**Figure 3:**
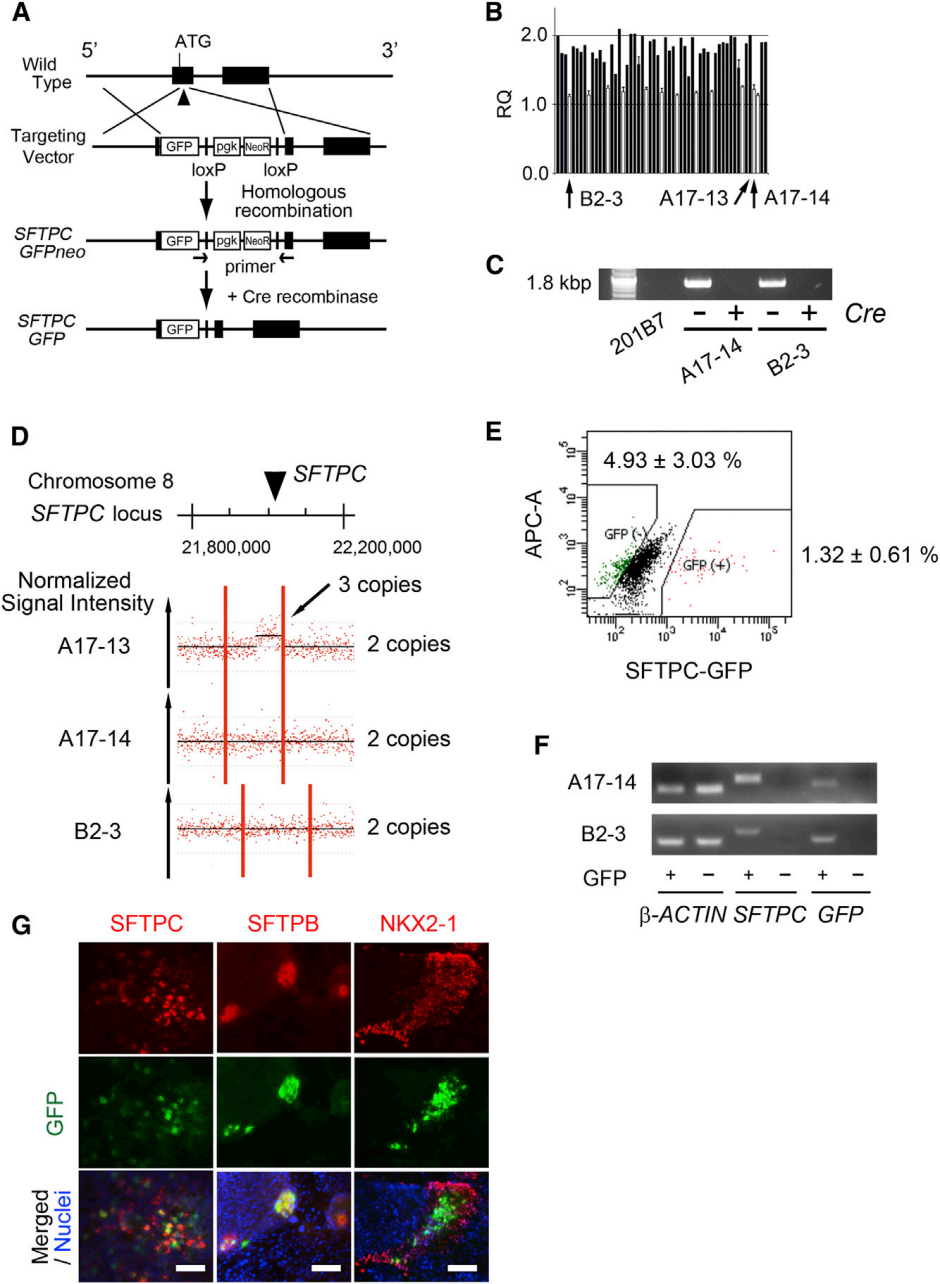
Generation of *SFTPC-GFP* Knockin hPSC Lines (A) Strategy for BAC-based gene targeting to produce *SFTPC-GFP* knockin hPSC lines. (B) Screening of knockin hPSC lines using TaqMan qPCR. Positive clones with candidate heterozygous deletion of the endogenous *SFTPC* gene are shown in white bars. Only clones that were initially suspected to be positive were tested independently three times. (C) Genomic PCR to confirm the removal of the *pgk-NeoR* cassette by Cre-recombinase in the A17-14 and B2-3 *SFTPC-GFP* reporter hPSCs. (D) SNP array analysis of the *SFTPC-GFP* knockin hPSC lines. The copy number of *SFTPC* gene loci was analyzed in A17-13, A17-14, and B2-3 clones. The A17-14 and B2-3 clones have two copies of the *SFTPC* gene loci, whereas the A17-13 clone has three copies of the loci. The red dots and y axis represent the normalized signal intensity of each SNP. (E) Isolation of SFTPC^−^GFP^+^ and GFP^−^ cells via FACS after sorting CPM^+^ cells via MACS on day 25. (F) RT-PCR analyses of GFP^+^ and GFP^−^ sorted cells in the A17-14 and B2-3 *SFTPC-GFP* reporter hPSC lines. (G) Representative images of GFP detected in SFTPC^+^, SFTPB^+^, and NKX2-1^+^ cells. Error bars show SEM. Scale bars, 100 μm. See also Figure S3 and Tables S1 and S2.

In order to investigate whether CPM is a potential surface marker of AEPCs, we generated *SFTPC-GFP* knockin reporter hPSC lines from 201B7 hiPSCs using BAC-based homologous recombination methods (Mae et al., 2013; Figure 3A; Supplemental Experimental Procedures), as SFTPC is the most specific marker of type II AECs. Following electroporation of the targeting vectors, 12 of 55 G418-resistant clones were found to have a heterozygous deletion of the genomic endogenous *SFTPC*-coding region (Figure 3B). The *pgk-Neo* cassette was removed via electroporation of the Cre-expression vector (Figure 3C), and normal karyotypes of the A17-14 and B2-3 clones were confirmed (Figure S3). The genomic copy number was calculated as previously described (Mae et al., 2013). The parental 201B7 (data not shown), A17-14, and B2-3 clones have two copies of the *SFTPC* gene loci, in contrast to the A17-13 clone, in which random transgenic integration is supposed to have occurred, as indicated by three copies of the loci (Figure 3D). No copy-number variation was detected for the B2-3 clone, whereas a copy-number loss at chromosome 16 q23.3 and gain at chromosome 20 p13 were detected for the A17-14 clone (data not shown). Both *SFTPC-GFP* reporter hPSCs were then differentiated to the end of step 5 and GFP^+^ and GFP^−^ cells were obtained by FACS after the CPM^+^ cells were sorted using MACS (Figure 3E). We confirmed the correlation between *GFP* and *SFTPC* on RT-PCR (Figure 3F). GFP was detected in SFTPC^+^, SFTPB^+^, and NKX2-1^+^ cells for both clones (Figure 3G).

### Alveolar Differentiation from CPM^+^ VAFECs in 3D Coculture

**Figure 4:**
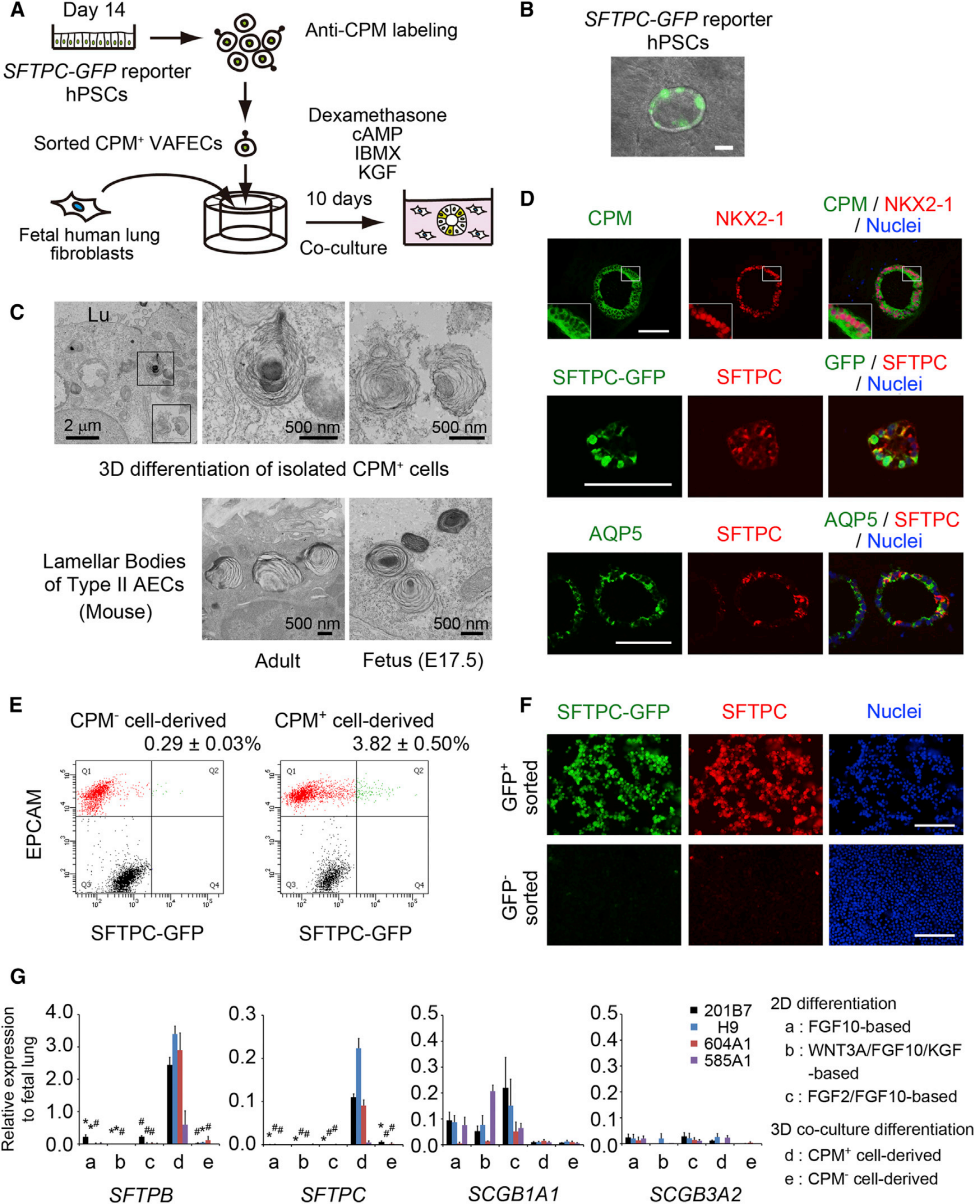
Alveolar Differentiation from CPM^+^ VAFECs in 3D Coculture (A) Strategy for inducing AECs via 3D coculture with FHLFs. (B) SFTPC-GFP^+^ cells detected in spheroids derived from isolated CPM^+^ VAFECs. (C) Transmission electron microscopy of lamellar-body-like structures observed in 3D coculture differentiation of CPM^+^ cells compared with those observed in the adult and fetal murine lungs. Lu, lumen. (D) IF staining of spheroids derived from CPM^+^ VAFECs. (E) Flow cytometry of SFTPC-GFP^+^ cells in 3D coculture differentiation of CPM^+^ cells or CPM^−^ cells (n = 3). (F) GFP^+^ and GFP^−^ cells isolated via FACS, spun down onto slides, and stained by anti-GFP and anti-SFTPC antibodies. (G) qRT-PCR comparing the 2D and 3D differentiation into AECs in H9 hESCs and 201B7 (parental), 604A1, and 585A1 hiPSCs. Each value of the gene expression was normalized to the level of *β-ACTIN*. The levels of the fetal lungs were set at one. Values are presented as the mean ± SEM. Error bars show SEM. #p < 0.05, ^∗^p < 0.01. Scale bars, 100 μm unless otherwise indicated. See also Figure S4 and Tables S1 and S2.

We attempted 2D differentiation, reseeding the CPM^+^ *SFTPC-GFP* reporter hPSCs purified from VAFECs on day 14 onto Matrigel-coated, 96-well plates. After 14 days of differentiation in step 5 medium, SFTPB became positive in the reseeded CPM^+^ cells (Figure S2E); however, *SFTPC* was almost negative (Figure S4D, condition b). We obtained similar results when we sorted and reseeded CPM^+^ cells on day 23 (Figure S2F). The discrepancy between the expression of SFTPB and SFTPC in developing human lungs was previously reported (Khoor et al., 1994). Therefore, we hypothesized that some missing factors are important for the coexpression of SFTPB and SFTPC. We then adopted a 3D coculture with fetal human lung fibroblasts (FHLFs) obtained at 17.5 weeks of gestation (Figure 4A). CPM^+^ cells purified from VAFECs on day 14 and FHLFs were mixed at a ratio of 1:50 and reseeded onto cell inserts. After 10 days of differentiation in step 5 medium, GFP became positive in some spheroids (Figure 4B). The spheroids were subsequently examined with a transmission electron microscope and lamellar-body-like structures were noted (Figure 4C). On hematoxylin-and-eosin staining, cyst-like spheroids consisting of pseudostratified, columnar, or cuboidal cells with dark pink cytoplasm were observed in the CPM^+^ cell-derived spheroids, whereas small pieces of spheroids consisting of cuboidal cells with clear cytoplasm were noted in the CPM^−^ cell-derived spheroids (Figure S4A). On IF staining, CPM and NKX2-1 were double positive in most CPM^+^ cell-derived spheroids, while GFP and SFTPC were double positive in some spheroids (Figure 4D). In the CPM^−^ cell-derived spheroids, EPCAM was positive, whereas no CPM^+^ or NKX2-1^+^ cells were identified (Figure S4B). SFTPA, SFTPB, SFTPC, and SFTPD (representative markers of type II AECs) were positive in the CPM^+^ cell-derived spheroids (Figure S4C). AQP5^+^ cells were adjacent to SFTPC^+^ cells in some spheroids (Figure 4D). ID2 and SOX9 (markers of differentiation into the distal lung-lineage fate) were positive in some NKX2-1^+^ and CPM^+^ cells, respectively (Figure S4C). Next, we trypsinized the cells in 3D structures and determined the proportion of SFTPC-GFP^+^ cells, detecting 3.82% ± 0.50% cells obtained from the CPM^+^ cell-derived 3D structures and 0.29% ± 0.03% cells obtained from the CPM^−^ cell-derived structures including fibroblasts (Figure 4E). Excluding the fibroblasts, the ratio of the number of SFTPC-GFP^+^ cells to that of EPCAM^+^ cells was calculated to be 9.81% ± 1.81% in the CPM^+^ cell-derived spheroids and 1.07% ± 0.16% in the CPM^−^ cell-derived spheroids. Almost all of the GFP^+^ cells sorted by FACS were SFTPC^+^, whereas the GFP^−^ cells were SFTPC^−^ (Figure 4F). The levels of alveolar markers (*SFTPB* and *SFTPC*), rather than club cell markers (*SCGB1A1* and *SCGB3A2*), were significantly elevated following the 3D coculture differentiation of CPM^+^ cells derived from three hPSC lines (H9 hESCs and parental 201B7 and 604A1 hiPSCs) compared with the 2D differentiation employing the three protocols separately starting on day 14 (Figure 1A; Green et al., 2011; Longmire et al., 2012) and the 3D coculture differentiation of CPM^−^ cells (Figure 4G). Interestingly, the levels of *SFTPB* and *SFTPC* were quite low for 585A1 hiPSCs, suggesting that the concentration of retinoic acid required to induce NKX2-1^+^ VAFECs in step 3 is less important for subsequent differentiation into AECs than the difference in the cell lines or donors. Moreover, the expression of *SFTPB* and *SFTPC* was small for the 2D and 3D differentiation of CPM^+^ cells alone or FHLFs alone (Figure S4D). Finally, other cell-type markers (*AQP5* [type I AECs], *FOXJ1* [ciliated cells], and *AGR2* [goblet cells]) appeared to be elevated in the CPM^+^ cell-derived structures rather than in the CPM^−^ cell-derived structures, suggesting that cell-type markers other than club-cell markers were expressed in the CPM^+^ cell-derived spheroids. *KRT5* (a basal cell marker, possibly including both airway and esophageal basal cells) was exclusively expressed in the CPM^−^ cell-derived structures. In addition, *PAX8* (a thyroid marker), *PAX6* (a neuron marker), and the other foregut endodermal lineage cells (*FOXN1*, *ALB*, and *PDX1*) were only minimally or slightly induced following 3D coculture differentiation (Figure S4E).

## Discussion

In this work, we identified CPM as a surface marker that is expressed in NKX2-1^+^ VAFECs, including AEPCs, and demonstrated that the CPM^+^ cell-derived spheroids obtained via 3D coculture differentiation with FHLFs enabled more efficient differentiation to AECs than did 2D differentiation. The gene-expression pattern of *CPM* in developing lungs has not received significant attention, although in situ hybridization of *Cpm* in anterior DECs as early as E7.5 in mice has been reported (Tamplin et al., 2008). Our data from IF staining of murine fetal lungs (Figure S1I) also suggest that lineage-tracing studies may provide answers to the following questions: Is Cpm a possible “specific” marker of lung-lineage progenitor cells such as Shh (Harris et al., 2006), Id2 (Rawlins et al., 2009a), and Nkx2-1 (Longmire et al., 2012)? What is the relationship between CPM^+^ cells and bipotent cells that are capable of generating type I and type II AECs (Desai et al., 2014)? Do CPM^+^ cells differentiate into type II AECs directly or indirectly via SFTPC^+^SCGB1A1^+^ cells (Kim et al., 2005; Rawlins et al., 2009b)? Furthermore, the present study suggests that a 3D microenvironment and coculture with FHLFs are important factors in the differentiation of progenitor cells into AECs rather than club cells. Although maintaining type II AECs in 2D conditions is often difficult (Dobbs, 1990; Yu et al., 2007), 3D conditions have recently been applied with better outcomes (Yu et al., 2007; McQualter et al., 2010; Barkauskas et al., 2013). Therefore, our 3D differentiation protocol appears to be a reasonable approach for maintaining differentiated type II AECs, although methods for expanding such cells for longer periods should be established in the next step.

The limitations of the present study include the fact that we were unable to demonstrate whether CPM is a more appropriate marker for lung-lineage cells than NKX2-1. Future studies focusing on the possible contribution of NKX2-1^−^CPM^+^ cells and/or NKX2-1^+^CPM^−^ cells to the differentiation of lung epithelial cells may resolve this issue, although we found only two isolatable populations of NKX2-1^+^CPM^+^ and NKX2-1^−^CPM^−^ cells using the present protocol. In addition, we were unable to demonstrate the highest induction efficiency of AECs, as recently described (Ghaedi et al., 2013), although we employed a different method for evaluating efficiency using *SFTPC-GFP* reporter hPSCs. Another limitation is that the functions of the induced AECs remain to be elucidated.

Nevertheless, the methods applied in the present study to induce and isolate AEPCs using CPM and consequently generate alveolar epithelial spheroids in a stepwise fashion may help to elucidate the complicated differentiation of human AECs and open the door for the development of new strategies for in vitro toxicology and cell replacement therapy, as well as screening for therapeutic drug compounds, in the future.

## Experimental Procedures

### 2D Differentiation

CHIR99021 (Axon Medchem), an activator of canonical Wnt signaling, was substituted for WNT3A (Mae et al., 2013). For details regarding the protocols used for each differentiation medium, see the Supplemental Experimental Procedures.

### 3D Differentiation

The protocol for the 3D culture was modified from a previous report (Barkauskas et al., 2013). For further details, see the Supplemental Experimental Procedures.

### Ethics

The use of H9 hESCs was approved by the Ministry of Education, Culture, Sports, Science and Technology (MEXT) of Japan. Human ethics approval was obtained from the Institutional Review Board and Ethics Committee of Kyoto University Graduate School and Faculty of Medicine. Animal ethics approval was obtained from the Animal Ethics and Research Committee of Kyoto University.

### Statistical Analysis

Values are expressed as the mean ± SEM and “n” stands for the number of independent experiments. Two-tailed Student’s t test was performed to identify significant differences between two conditions of qRT-PCR.

## Author Contributions

S.G., I.I., and K.O. designed the study. S.G., T.N., Y.Y., S.K., Y.K., and A.S.-O. performed the experiments. S.G., I.I., T.N., Y.Y., S.K., A.S.-O., S.O., and K.O. analyzed the data. S.G. and I.I. wrote the manuscript through fruitful discussions with and supervision by H.M., S.M., T.H., S.O., K.O., and M.M. M.F., S.-I.M., T.T., and K.O. provided the method for inducing definitive endoderm and advised on the methods used for vector construction and other basic techniques.

## Accession Numbers

The NCBI GEO accession number for the microarray data reported in this paper is GSE53513.

## Supplemental Information

Document S1. Supplemental Experimental Procedures, Figures S1–S4, and Tables S1 and S2

Document S2. Article plus Supplemental Information
