# Supplementary material for: Generation of Alveolar Epithelial Spheroids via Isolated Progenitor Cells from Human Pluripotent Stem Cells
Source: Stem Cell Reports. 2014 Aug 21;3(3):394–403. doi: 10.1016/j.stemcr.2014.07.005 (PMC4266003; doi:10.1016/j.stemcr.2014.07.005)
Supplement: Document S1. Supplemental Experimental Procedures, Figures S1–S4, and Tables S1 and S2 [file mmc1.pdf]

Stem Cell Reports, Volume *3*

Supplemental Information

Generation of Alveolar Epithelial Spheroids via Isolated Progenitor Cells from Human Pluripotent Stem Cells

Shimpei Gotoh, Isao Ito, Tadao Nagasaki, Yuki Yamamoto, Satoshi Konishi, Yohei Korogi, Hisako Matsumoto, Shigeo Muro, Toyohiro Hirai, Michinori Funato, Shin-Ichi Mae, Taro Toyoda, Aiko Sato-Otsubo, Seishi Ogawa, Kenji Osafune, and Michiaki Mishima

Figure S1, related to Figure 1.

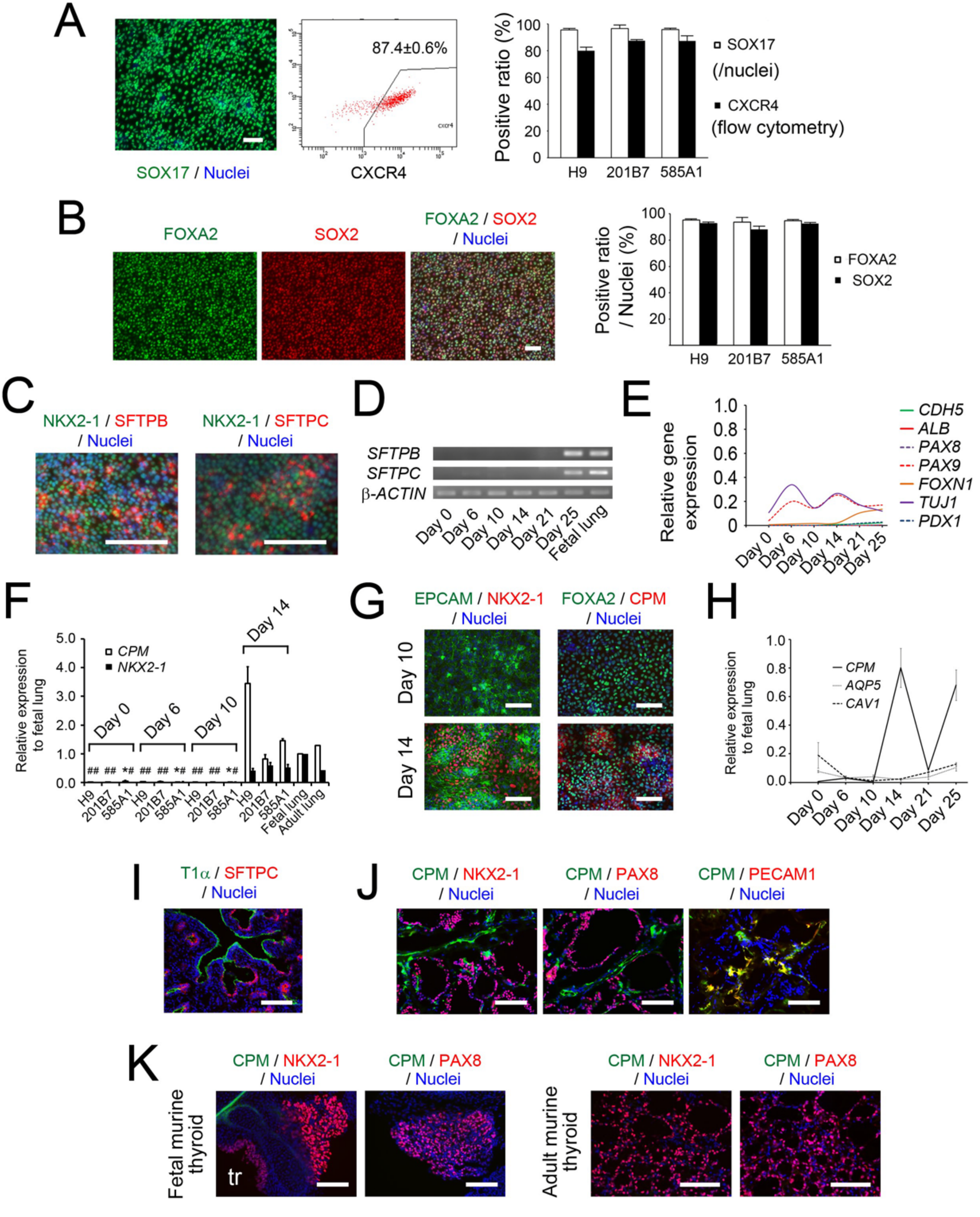

Figure S2, related to Figure 2.

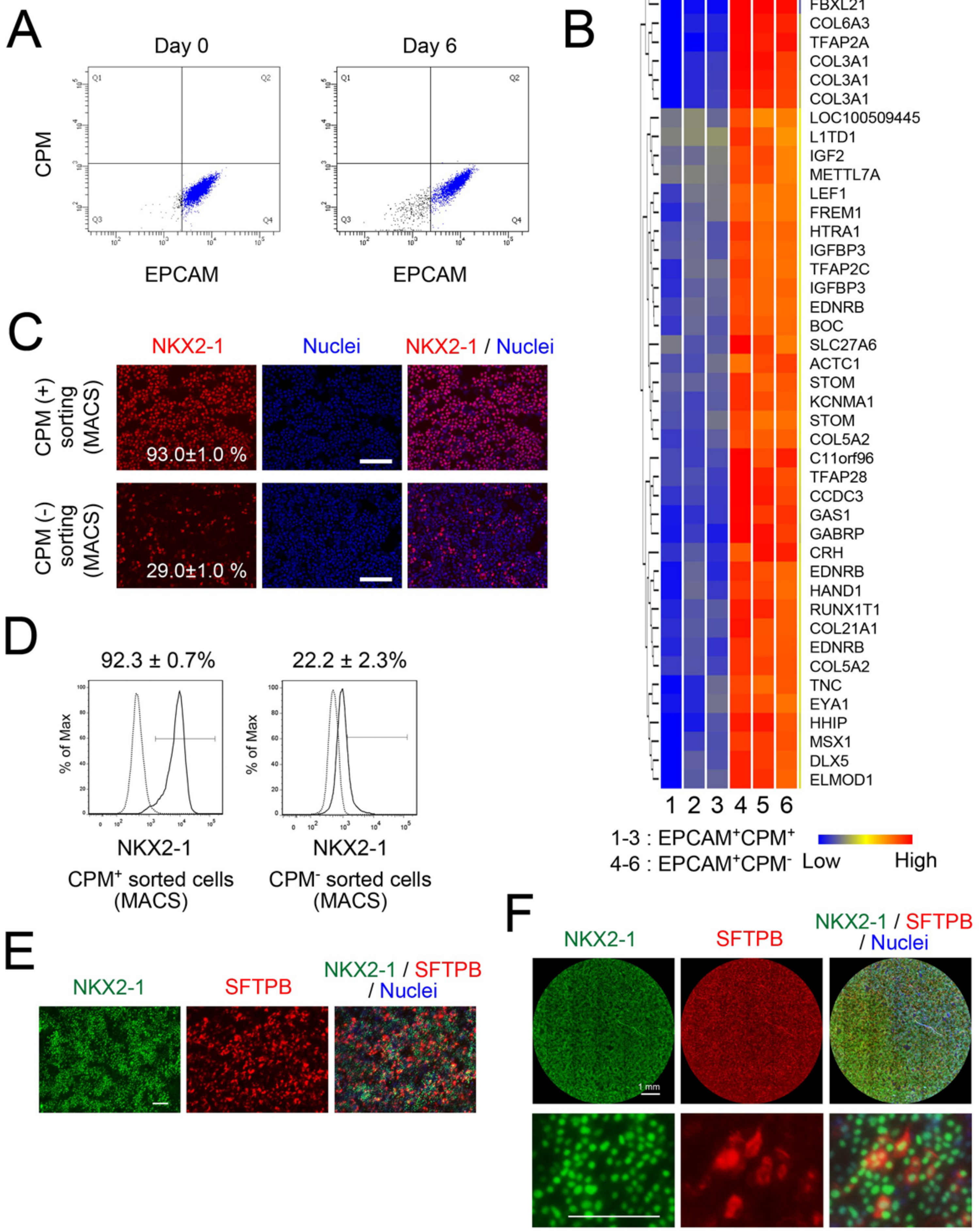

Figure S3, related to Figure 3.

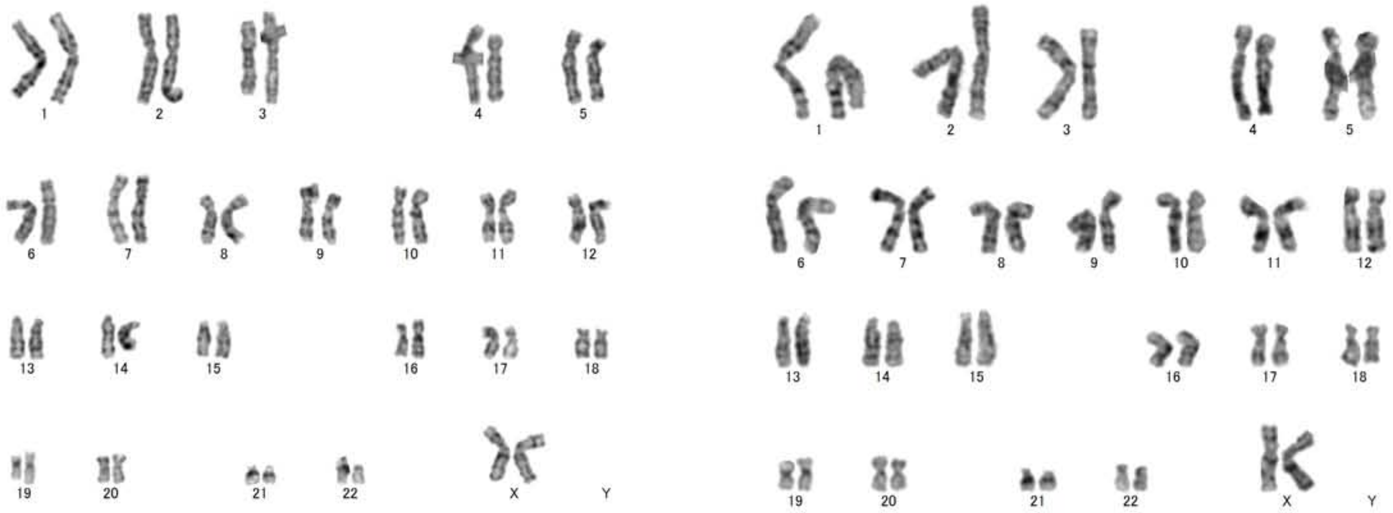

A17-14 B2-3

Figure S4, related to Figure 4.

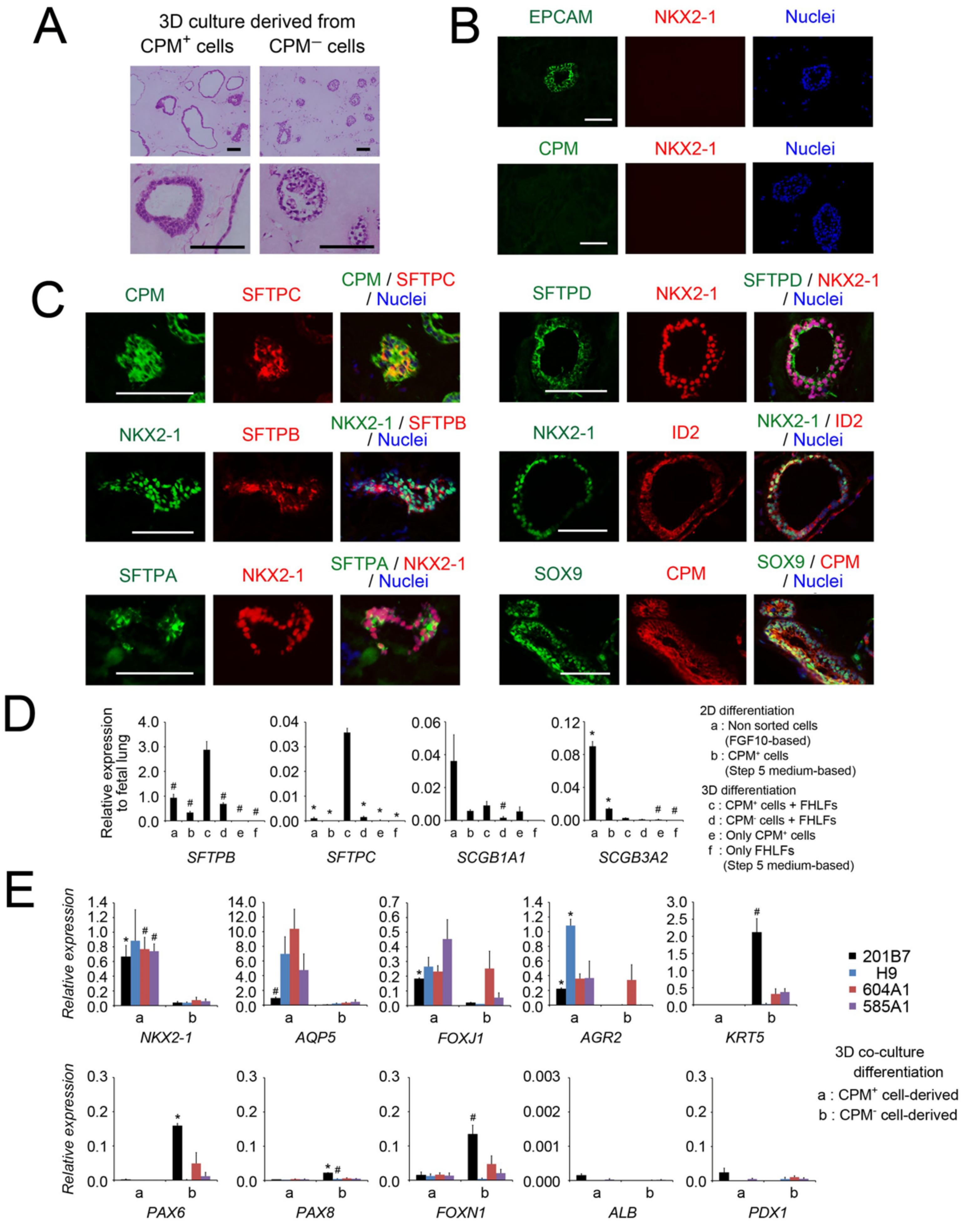

Table S1, related to Figure 1, 2, 3 and 4. Primers used in the present study.

Gene name		Primer Sequence	Size (bp)
β-ACTIN	F	CAATGTGGCCGAGGACTTTG	126
	R	CATTCTCCTTAGAGAGAAGTGG	
SOX17	F	CGCTTTCATGGTGTGGGCTAAGGACG	186
	R	TAGTTGGGGTGGTCCTGCATGTGCTG	
GATA6	F	CAGCAAAAATACTTCCCCCA	107
	R	ACTTGAGCTCGCTGTTCTCG	
FOXA2	F	TCGCTCTCCTTCAACGACTGTTTCC	107
	R	TTCTCGAACATGTTGCCCGAGTCAG	
SOX2	F	AGTCTCCAAGCGACGAAAAA	189
	R	TTTCACGTTTGCAACTGTCC	
NKX2-1	F	AGCACACGACTCCGTTCTC	68
	R	GCCCACTTTCTTGTAGCTTTCC	
CPM	F	TCCAAGGTGGAATGCAAGAT	181
	R	TCAAAAACTTGACCCTTTACACC	
SOX9	F	GAGGAAGTCGGTGAAGAACG	337
	R	ATCGAAGGTCTCGATGTTGG	
ID2	F	GACAGCAAAGCACTGTGTGG	144
	R	CCATTCAACTTGTCCTCCTTG	
HOPX	F	TCAACAAGGTCGACAAGCAC	157
	R	TCTGTGACGGATCTGCACTC	
SCGB1A1	F	TTCAGCGTGTCATCGAAACCC	189
	R	ACAGTGAGCTTTGGGCTATTTTT	
SCGB3A2	F	CAAGTGGAACCACTGGCTTG	198
	R	CCAGAGGTAAAGGTGCCAAC	
SFTPA2	F	AAGCAGCTGGAGGCTCTGT	88
	R	CCATCAAGATGAGGGTGAGG	
SFTPB	F	GAGCCGATGACCTATGCCAAG	133
	R	AGCAGCTTCAAGGGGAGGA	
SFTPC	F	GCAAAGAGGTCCTGATGGAG	178
	R	TGTTTCTGGCTCATGTGGAG	
DCLAMP	F	ACCGATGTCCAACTTCAAGC	161
	R	TGACACCTTAGGCGGATTTT	
AQP5	F	CTGTCCATTGGCCTGTCTGTC	248
	R	GGCTCATACGTGCCTTTGATG	
CAV1	F	AGGGCAACATCTACAAGCCC	188
	R	GCCGTCAAAACTGTGTGTCC	
NGFR	F	CGACAACCTCATCCCTGTCT	102
	R	TTGTTCTGCTTGCAGCTGTT	
KRT5	F	GAGCTGAGAAACATGCAGGA	82
	R	CAAGCGTACCACTGCTGAGA	
AGR2	F	AGCACTAGTGGGTGGGATTG	167
	R	GCAAGAATGCTGACACTGGA	
FOXJ1	F	CCTGTCGGCCATCTACAAGT	94
	R	AGACAGGTTGTGGCGGATT	
TUJ1	F	GCAACTACGTGGGCGACT	86
	R	TCGAGGCACGTACTTGTGAG	
PAX6	F	CGGAGTGAATCAGCTCGGTG	301
	R	CCGCTTATACTGGGCTATTTTGC	
PAX8	F	TCAACCTCCCTATGGACAGCTG	137
	R	GAGCCCATTGATGGAGTAGGTG	
PAX9	F	TGGTTATGTTGCTGGACATGGGTG	136
	R	GGAAGCCGTGACAGAATGACTACCT	
FOXN1	F	TGGAGAGTGGTGCTGGGATGTT	141
	R	GGTACTGATAGTGTGAGGAGCC	
CDH5	F	ACACCTCACTTCCCCATCA	95
	R	GACCTTGCCCACATATTCTCC	
PDX1	F	CCCATGGATGAAGTCTACC	262
	R	GTCCTCCTCCTTTTTCCAC	
ALB	F	CCTTTGGCACAATGAAGTGGGTAACC	164
	R	CAGCAGTCAGCCATTTCACCATAG	
EGFP	F	AGAACGGCATCAAGGTGAAC	135
	R	TGCTCAGGTAGTGGTTGTCG	

Table S2, related to Figure 1, 2, 3 and 4. Antibodies used in the present study.

Primary Antibodies	Dilution rate	Manufacturer	Clone / Cat. No.
GFP	1:500	Aves Labs	GFP-1020
SFTPA	1:100	Immuno-Biological Laboratories	PE10 / 10375
proSPB / SFTPB	1:2000	EMD-Millipore	AB3432
proSPC / SFTPC	1:2000	EMD-Millipore	AB3786
SFTPC	1:100	Santa Cruz Biotechnology	sc-13979
SFTPD	1:500	Yamasa corporation	10H11 /#7608
NKX2-1	1:500	Novus Biologocals	EP1584Y / NB100-80062
NKX2-1	1:500	Lab Vision	8G7G3/1 / MS-699-P
SCGB3A2	1:2000	a kind gift of Dr. Shioko Kimura	
CPM	1:500	Leica microsystems	1C2 / NCL-CPMm
CPM	1:250	Medical & Biological Laboratories	D293-3
AQP5	1:100	Santa Cruz Biotechnology	sc-9890
T1a / Podoplanin	1:100	Santa Cruz Biotechnology	18H5 / sc-59347
PECAM	1:100	Santa Cruz Biotechnology	sc-1506
EPCAM	1:100	Santa Cruz Biotechnology	EBA-1 / sc-66020
EPCAM (FITC)	1:10	BD Biosciences	EBA-1 / 347197
SOX17	1:500	R&D systems	AF1924
CXCR4 (PE)	1:10	R&D systems	12G5 / FAB170P
FOXA2	1:500	R&D systems	AF2400
SOX2	1:500	EMD-Millipore	AB5603
SOX9	1:20	R&D systems	AF3075
ID2	1:100	Santa Cruz Biotechnology	sc-489
KRT5	1:100	Lab Vision	EP1601Y / RM-2106-S0
PAX8	1:500	Proteintech	10336-1-AP
Mouse isotype control IgG1	1:100	Sigma-Aldrich	MOPC21 / M5284
Rabbit isotype control IgG	1:1000	Cell Signaling Technology	DA1E / #3900
Mouse isotype control IgG1 (FITC)	1:10	BD Biosciences	555748

Secondary Antibodies	Dilution rate	Manufacturer	Cat. No.
Donkey anti-mouse (Alexa546)	1:500	Life Technoloies	A10036
Donkey anti-mouse (Alexa488)	1:250	Life Technoloies	A21202
Goat anti-mouse (Alexa647)	1:200	Life Technoloies	A21236
Donkey anti-goat (Alexa488)	1:500	Life Technoloies	A11055
Donkey anti-goat (Cy3)	1:500	Jackson ImmunoResearch	705-165-147
Donkey anti-chicken (DyLight 488)	1:500	Jackson ImmunoResearch	703-485-155
Donkey anti-rabbit (Cy3)	1:500	Jackson ImmunoResearch	711-165-152
Donkey anti-rat (Alexa488)	1:500	Life Technoloies	A21208
Rat anti-mouse IgG1 (microbeads)	1:5	Miltenyi Biotec	130-047-101
Zenon Alexa488 Mouse IgG1 Labeling Kit	Manufacturer's protocol	Life Technoloies	Z-25002
Zenon Alexa555 Mouse IgG1 Labeling Kit	Manufacturer's protocol	Life Technoloies	Z-25005
Zenon Alexa647 Mouse IgG1 Labeling Kit	Manufacturer's protocol	Life Technoloies	Z-25008

SUPPLEMENTAL FIGURE LEGENDS

Figure S1. Related to Figure 1.

(A) Definitive endoderm cells (DECs) on Day 6. The efficiency of induction was examined by scoring the number of $SOX17^+$ cells relative to the total number of nuclei in an average of 10 randomly selected images (n=3) and counting the number of $CXCR4^+$ cells using flow cytometry. (B) Anterior foregut endoderm cells (AFECs) on Day 10. The efficiency was examined by scoring the number of $FOXA2^+SOX2^+$ cells relative to the total number of nuclei. (C) SFTPB and SFTPC detected in NKX2-1^+ cells on Day 25, respectively. (D) Electrophoresis of the RT-PCR products. Both *SFTPB* and *SFTPC* were positive only on Day 25 and in the positive control of the fetal human lungs. (E) RT-qPCR of *CDH5*, *ALB*, *PAX8*, *PAX9*, *FOXN1*, *TUJ1* and *PDX1*. Each gene expression level was normalized to that of *β-ACTIN* (n=3). The levels of positive controls in the fetal human lungs (*CDH5*), liver (*ALB*), thyroid (*PAX8*) and throat (*PAX9*), postnatal thymus (*FOXN1*) and the adult brain (*TUJ1*) and pancreas (*PDX1*) were set at 1. (F) RT-qPCR of *CPM* and *NKX2-1*. The levels of *CPM* and *NKX2-1* significantly increased from Day 0 to Day 14 in the H9, 201B7, and 585A1 cell lines (n=3). The gene expression observed in the fetal lungs was set at 1. (G) Expression of EPCAM and FOXA2 double-stained with NKX2-1 or CPM, respectively, on

Day 10 and Day 14. (H) Levels of *CPM* and other markers of type I AECs, including *AQP5* and *CAV1*, from Day 0 to Day 25 in the 201B7 cells (n=3). (I) T1α and SFTPC stained in the fetal human lung. (J) CPM in the adult human thyroid. CPM was not detected in NKX2-1^{+} cells or PAX8^{+} cells, although it was detected in PECAM1^{+} cells. (K) Negative expression of CPM in NKX2-1^{+} cells or PAX8^{+} cells in the fetal murine thyroid (E17.5) or adult murine thyroid (20 weeks). CPM was slightly positive in the perichondrium of the tracheal cartilage in the fetal mice. Tr: trachea.

The values are presented as the mean ± SEM. # $p < 0.05$. * $p < 0.01$. Scale bars, 100 μm.

Figure S2. Related to Figure 2.

(A) Flow cytometry of CPM^{+} and EPCAM^{+} cells on Day 0 and Day 6. The number of CPM^{+} cells was almost zero for both samples (n=3). (B) Cluster of genes decreased in the EPCAM^{+}CPM^{+} cells compared with that observed in the EPCAM^{+}CPM^{-} cells (n=3). (C) NKX2-1^{+} cells in MACS-sorted CPM^{+} and CPM^{-} cells derived from VAFECs. The proportion of NKX2-1^{+} cells was analyzed by scoring the number of NKX2-1^{+} cells relative to the total number of nuclei in an average of five randomly selected images (n=3). (D) Flow cytometry of NKX2-1^{+} cells in MACS-sorted CPM^{+} and CPM^{-} cells derived from

VAFECs on Day 14. Black line: anti-NKX2-1 antibody. Gray dotted line: isotype control (n=3). (E) Reseeded CPM^+ cells isolated on Day 14 and cultured in Step 5 medium in 2D for 14 days. The cells expressed NKX2-1 and SFTPB. (F) Whole-well imaging of the reseeded CPM^+ cells isolated on Day 23 and cultured in Step 5 medium in 2D for 14 days. The cells expressed NKX2-1 and SFTPB ubiquitously.

The values are presented as the mean ± SEM. Scale bars, 100 μm.

Figure S3, Related to Figure 3.

Normal karyotypes of the *SFTPC-GFP* reporter hPSC lines (A17-14 and B2-3).

Figure S4, Related to Figure 4.

(A) Hematoxylin-eosin staining of spheroids derived from CPM^+ cells and CPM^- cells in VAFECs. Both cell populations formed spheroids with an intraluminal space. (B) CPM^- cell-derived spheroids expressing EPCAM. (C) Expression of various markers of AECs in the CPM^+ cell-derived spheroids. (D) RT-qPCR comparing the 2D and 3D differentiation into AECs in the B2-3 *SFTPC-GFP* knock-in hPSC line (n=3). Each value was normalized to the level of *β-ACTIN*. The gene expression level observed in the fetal lungs was set at 1.

FHLFs: fetal human lung fibroblasts. (E) RT-qPCR comparing CPM^+ and CPM^- cell-derived 3D co-culture differentiation in H9 hESCs and 201B7 (parental), 604A1 and 585A1 hiPSCs for *NKX2-1*, *AQP5*, *FOXJ1*, *AGR2*, *KRT5*, *PAX6*, *PAX8*, *FOXN1*, *ALB* and *PDX1* (n=3). The levels of positive controls in the fetal human lungs (*NKX2-1*, *AQP5*, *FOXJ1*, *AGR2* and *KRT5*), thyroid (*PAX8*) and liver (*ALB*), postnatal thymus (*FOXN1*) and the adult brain (*PAX6*) and pancreas (*PDX1*) were set at 1.

The values are presented as the mean ± SEM. # $p < 0.05$. * $p < 0.01$. Scale bars, 100 μm.

Table S1, Related to Figure 1, 2, 3 and 4. Primers used in the present study.

Table S2, Related to Figure 1, 2, 3 and 4. Antibodies used in the present study.

SUPPLEMENTAL EXPERIMENTAL PROCEDURES

Culture of hPSCs

hESCs and hiPSCs (H9, 201B6, 201B7, 253G1, 585A1, 604A1 and 648A1) were cultured on feeder cells of mitomycin C-treated STO cells in Primate ES medium (ReproCell) supplemented with 50 U/ml of penicillin/streptomycin (Life Technologies) and 4 ng/ml recombinant human basic fibroblast growth factor (Wako). Passaging was performed using CTK dissociation solution containing 0.25% trypsin (Life Technologies), 0.1% collagenase IV (Life Technologies), 20% KSR (Life Technologies) and 1mM of $CaCl_2$ in PBS with a split ratio of 1:2 or 1:3.

2D differentiation

When the hPSCs reached 70% confluence (Day 0), the cells were incubated in 10 μM of Y-27632 (Wako) for one hour followed by deprivation of STO feeder cells with CTK dissociation solution. The cells were subsequently rinsed carefully with PBS and incubated in Accutase (Innovative Cell Technologies) for 20 minutes at 37°C. The detached hPSCs were then dissociated into single cells via pipetting, incubated on a 0.1% gelatin-coated plate for 30 minutes at 37°C and seeded on Matrigel-coated plates (BD Biosciences) at a

density of 1.1 x10^5 cells/cm^2 in the Step 1 medium containing RPMI1640 medium (Nacalai Tesque), 1x B27 supplement (Life Technologies, #17504-044) as the basal medium in addition to 50 U/ml of penicillin/streptomycin, 100 ng/ml of human activin A (R&D systems) and 1 μM of CHIR99021 (Axon Medchem), supplemented with 10 μM of Y-27632 (Day 0) and 0.25mM (Day 1) and 0.125mM (Day 2-6) of sodium butyrate (Kajiwara et al., 2012).

From Step 2 to Step 4, the basal medium consisted of DMEM/F12 plus Glutamax (Life Technologies), 1x B27 and N2 supplements (Life Technologies, #17502-048), 50 U/ml of penicillin/streptomycin, 0.05 mg/ml of L-ascorbic acid (Sigma-Aldrich), 0.4 mM of monothioglycerol (Wako), as previously reported (Green et al., 2011).

On Day 6, the medium was changed to Step 2 medium, containing the basal medium with 100 ng/ml of human recombinant noggin (R&D systems) and 10 μM of SB-431542 (R&D systems) (Green et al., 2011). On Day 10, the medium was changed to Step 3 medium, containing the basal medium with 100 ng/ml of human recombinant BMP4 (Humanzyme), 0.05-1.0 μM of all-trans retinoic acid (ATRA) (Sigma-Aldrich) and 1.5-3.5 μM of CHIR99021. The optimal concentration of ATRA/CHIR99021 was 0.5 μM/3.5 μM for H9 hESCs, 0.05 μM/2.5 μM for 201B7 hiPSCs, 0.05 μM/2.5 μM for 253G1 hiPSCs,

0.05 μM/3.5 μM for 201B6 hiPSCs, 0.5 μM/3.5 μM for 585A1 hiPSCs, 1.0 μM/2.5 μM for 604A1 hiPSCs, and 1.0 μM/2.5 μM for 648A1 hiPSCs, respectively.

On Day 14, the medium was changed to Step 4 medium in each protocol. For FGF10-based 2D differentiation (Figure 1), the medium was changed to that containing the basal medium with 100 ng/ml of human recombinant FGF10 (Wako).

On Day 21, the medium was changed to Step 5 medium consisting of Ham's F12 (Wako, #087-08335), 50 nM of dexamethasone (Sigma-Aldrich), 0.1 mM of 8-Br-cAMP (Biolog Life Science Institute, #B007), 0.1 mM of 3-Isobutyl-1-methylxanthine (IBMX) (Wako), 100 ng/ml of KGF (Wako), 0.25% of BSA (Life Technologies), 15 mM of HEPES (Sigma-Aldrich), 0.8 mM of $CaCl_2$ (Nacalai Tesque), 0.1% ITS premix (BD Biosciences), 50 U/ml of penicillin/streptomycin (Longmire, et al., 2012).

For WNT3A/FGF10/KGF-based 2D differentiation (Figure 4G and S4D), the medium was changed on Day 14 to that containing the Step 4 basal medium with 100 ng/ml of human recombinant WNT3A (R&D systems), 10 ng/ml of FGF10, and 10 ng /ml of KGF for eight days. (Green et al., 2011).

For FGF2/FGF10-based 2D differentiation (Figure 4G and S4D), the medium was changed on Day 14 to that containing the Step 4 basal medium with 500 ng/ml of basic

FGF and 100 ng/ml of FGF10 for seven days, followed by Step 5 medium for three days (Longmire et al., 2012). Each medium was replaced every two days throughout the differentiation process.

Flow cytometry

The cells were incubated in 10 μM of Y-27632 for one hour, followed by dissociation with Accutase for 20 minutes at 37°C. The detached cells were diluted in DMEM/F12 (Life Technologies) with 2% FBS and centrifuged at 800 rpm at room temperature (RT). The pellets of the cells were immersed with 1%BSA/PBS and centrifuged again. The samples were incubated in the primary antibodies for 15 minutes, washed twice with 1%BSA/PBS, and if necessary, incubated in the secondary antibodies for 15 minutes. After rinsing with 1% BSA/PBS twice, the cells were analyzed. The measurements were obtained using a BD FACSAria II flowcytometer (BD Biosciences). Isotype controls were used for gating CPM^+ or $EPCAM^+$ cells, whereas negative control cells that do not express GFP were used for gating GFP^+ cells.

Isolation of CPM^+ cells from VAFECs.

Induced VAFECs were washed twice with PBS supplemented with 0.5 mM of EDTA and dissociated with Accutase at 37°C for 20 minutes. After resuspending the detached cells in four times the volume of DMEM/F12 containing 2% FBS, the cell clumps were removed using a cell strainer with a 40-μm pore size (BD falcon).

For fluorescence-activated cell sorting (FACS), the dissociated cells were washed in 1% BSA/PBS, centrifuged at 800 rpm for five minutes, resuspended and incubated in mouse anti-human CPM antibody (0.4 μl/1.0×10^6 cells, diluted to 1:200 in 1% BSA/PBS) (Leica Microsystems) labeled with the Alexa 647-conjugated Fab fragment (Life Technologies) and FITC-conjugated mouse anti-human EpCAM antibody (BD Biosciences) at 4°C for 15 minutes. After rinsing with 1% BSA/PBS twice, the $EpCAM^+CPM^+$ and $EpCAM^+CPM^-$ cells were sorted using the BD FACSAria II flowcytometer (BD Biosciences).

For magnetic activated cell sorting (MACS), the dissociated cells were resuspended and incubated in mouse anti-human CPM antibody solution at 4°C for 15 minutes. After washing twice with 0.5% BSA/PBS with 2mM EDTA, the labeled cells were incubated in anti-mouse IgG1 microbeads solution (20 μl/1×10^7 cells, diluted to 1:4 in 0.5% BSA/PBS with 2mM EDTA) at 4°C for 15 minutes. After washing twice with the same

buffer, the CPM^+ cells were separated using a magnetic stainless column (Miltenyi Biotec). To improve purity, separation was performed twice, and the number of harvested cells was counted. 10 μM of Y-27632 was supplemented for all processes of FACS and MACS until sorting of the CPM^+ and CPM^- cells was completed.

3D differentiation

A total of $2.0x10^4$ CPM^+ cells isolated from induced VAFECs on Day 14 were mixed with $1.0x10^6$ of fetal human lung fibroblasts (17.5 weeks of gestation, DV Biologics, # PP002-F-1349, Lot.121109VA) in 1:1 Matrigel/Step 5 medium, and a total volume of 400 μl was seeded on a 12-well cell culture insert (BD Biosciences, #353180) with 10 μM of Y-27632 and 1 ml of Step 5 medium placed in the lower chamber. The cells were maintained by changing the medium in the lower chamber every other day for 10 days. For the FACS analyses, Matrigel blocks containing differentiated cells and fibroblasts were minced into small pieces and incubated in 0.1% Trypsin/0.5 mM EDTA at 37°C for 15 minutes with occasional pipetting.

Quantitative RT-PCR

Total RNA was isolated using the PureLink RNA Mini Kit (Life Technologies) according to the manufacturer's manual. First-strand cDNA was synthesized from 80 ng of total RNA using the SuperScript III First-Strand Synthesis System (Life Technologies). The cDNA samples were amplified using Power SYBR Green PCR Master Mix with ABI7300 Real-Time PCR System (Life Technologies). All reactions were started at a cycle of 95°C for 10 minutes, followed by 45 cycles of 95°C for 15 seconds, 62°C for 20 seconds and 72°C for 30 seconds. The PCR reactions were performed in triplicate for each sample. The level of expression of each gene was calibrated to that of the housekeeping gene, *β-ACTIN*, and compared to the level of the expression of each gene in the fetal human lungs (17, 18, and 22 weeks gestation, Agilent Technologies, #540177, Lot.0006055802). All primer sets are shown in Table S1.

Immunofluorescence staining

The tissue cryosections and cells were fixed with 4% paraformaldehyde /PBS (Nacalai Tesque) for 15 minutes at RT. After washing three times with PBS, the cells were immersed in 0.2% Triton X-100 /PBS for 15 minutes at RT, followed by incubation with the blocking solution consisting of 5% normal donkey serum (Millipore), 1% BSA

(Sigma-Aldrich), and PBS (Nacalai Tesque) for 30 minutes at RT. The cells were incubated in the primary antibody solution for 30 minutes at RT, followed by washing three times with 1% BSA/PBS, after which they were incubated in the secondary solution for 30 minutes at RT and washed three times with PBS. Regarding the results of staining for GFP and SFTPC, we concerned about following things. First, we avoided freezing the spheroids embedded in the OCT compounds (Sakura Finetek) before incubation with the primary anti-GFP (Aves Labs) and anti-proSPC antibodies (EMD-Millipore) in order to detect the gene expression in the spheroids (Figure 4D), due to the potential loss of antigenicity caused by freezing. Instead, whole-mount fixation and staining were performed before freezing and sectioning. Second, we also avoided use of anti-proSPC antibody (EMD-Millipore) to stain the cells in the two-dimensional culture, as nonspecific staining inconsistent with the results of RT-qPCR of *SFTPC* was observed. Indeed, nonspecific reactions of other commercial anti-proSPC antibody to cells in two-dimensional culture have recently been reported (Schmeckebier et al., 2013). Therefore, anti-SFTPC antibody (Santa Cruz Biotechnology) were used for the two-dimensional culture and staining of other tissue/spheroid sections. Corresponding gene expression analyses of *SFTPC* were performed using RT-qPCR. All primary and secondary antibodies used in the present study

were diluted in blocking solution as indicated in Table S2. Nuclei were counterstained with Hoechst-33342.

Generation of SFTPC-reporter knock-in hPSCs

The human BAC clones of RP11-102O8 and CTD-2530N21 both of which contains all of the exons of the *SFTPC* gene and extends from 138.1 kb upstream to 39.3 kb downstream of the gene locus for the former clone and 92.9 kb upstream to 123.8 kb downstream for the latter clone, were purchased from Life Technologies. Gene targeting was performed as previously described (Mae, et al., 2013). In order to construct the knock-in vector, we designed two primers with 60 bp sequences of the homologous recombination regions of the *SFTPC* gene with the 5' or 3' end of the *EGFP-pA-PNL* sequence (kindly provided by Dr. Kazutoshi Takahashi), and performed genomic PCR using KOD Plus Neo polymerase (Toyobo) according to the manufacturer's protocol. The primers used for genomic PCR were as follows: hSFTPC-EGFP-F, ATATAAGACCCTGGTCACACCTGGGAGAGGAGGAGAGGAGAGCATAGCACCTG CAGCAAGATGGTGAGCAAGGGCGAGGA; hSFTPC-PNL-R, CCCATCACACACATG TGCGCGCGCACACATACATACACACACGCAACCACACTCACCGGCGTCGACGGC

GAGCTCAGACG. The targeting vector, the *EGFP-pA-PNL* cassette containing 5' and 3' homology arms, was then electroporated into DH10B containing BAC RP11-102O8 and CTD-2530N21, respectively, and activated recombinases using the Red/ET-mediated recombination technique (Gene Bridges). The transformed bacteria were plated on LB plates with appropriate antibiotics and incubated overnight at 37°C. Selected clones were subjected to PCR to confirm whether the *EGFP-pA-PNL* cassette was integrated into the *SFTPC* endogenous locus via successful homologous recombination. The RP11-102O8 derived-targeting vector was named A17 and the CTD-2530N21 derived-targeting vector was named B2. The human *SFTPC-EGFP-pA-PNL* BAC vector was electroporated into hiPSCs (201B7), and the electroporated cells were plated on feeder layers of mitomycin C-treated STO cells. G418 antibiotic selection was applied two days after electroporation until each colony was picked up manually. In order to select positive clones in which homologous recombination occurred, real-time PCR reactions were carried out with 100 ng of genomic DNA using the predesigned TaqMan copy number assay (Assay ID: SFTPCdel_CCCSUW0). Among 55 G418-resistant clones, 12 clones were selected with a heterozygous deletion of the endogenous *SFTPC*-coding region replaced by the *GFP-pgk-Neo* cassette (Figure 3B). Because many candidate clones were obtained, two

clones, B2-3 and A17-14, were chosen for use in the subsequent studies. The hiPSCs with a targeted *SFTPC* allele were treated with 10 μM of Y27632 overnight and trypsinized. Cells resuspended in PBS were electroporated with 30 μg of pCXW-Cre-Puro (kindly provided by Dr. Keisuke Okita) and plated on feeder layers of mitomycin C-treated puromycin-resistant STO cells (kindly provided by Dr. Kazutoshi Takahashi). Antibiotic selection with 1 μg/ml of puromycin was applied two days after electroporation for two days. Conventional PCR was performed to confirm the loss of the *PGK-Neo* cassette. G-band analyses were carried out at Nihon Gene Research Laboratories, Japan. Genomic DNA was analyzed using CytoScan HD arrays (Affymetrix) according to the manufacturer's protocol. The genomic copy numbers were calculated using the CNAG3.5.1 software program (Nannya et al., 2005; Yamamoto et al., 2007). Genetic lesions, including copy number gains and losses, were detected using a hidden Markov model (HMM)-based algorithm implemented in the CNAG3.5.1 software program (http://www.genome.umin.jp/CNAG_DLpage/CNAG_top.html). RT-PCR of *GFP* and *SFTPC* were performed as follows: initial denaturation was 95°C for 10 minutes, followed by 25 cycles (*β-ACTIN*) or 30 cycles (*GFP* or *SFTPC*) of 95°C for 15 seconds, 62°C for 20 seconds and 72°C for 30 seconds.

Gene expression studies using microarrays

Biotinylated cRNA was synthesized using the GeneChip 3'IVT Express Kit (Affymetrix) from 250 ng of total RNA according to the manufacturer's instructions. A total of 10 ug of cRNA was hybridized for 16 hours at 45°C on the GeneChip Human Genome U133 Plus 2.0 Array, followed by washing and staining in the Affymetrix Fluidics Station 450. The GeneChips were scanned using the GeneChip Scanner 3000 7G, and the resulting images were quantified and normalized according to the MAS5.0 algorithm. The trimmed mean target intensity of each chip was arbitrarily set to 500. The data were processed using Gene Spring GX software program.

Electron microscopy

3D matrices involving differentiated cells were cut into small pieces and incubated in fixative containing 2.5% glutaraldehyde, 2% paraformaldehyde, 2% osmium tetroxide, 0.1% picric acid, 4% sucrose, and 0.1M phosphate buffer (pH 7.4) at 4°C for two hours, followed by incubation in 1% uranyl acetate en bloc at RT for one hour (Osanai et al., 2010). The samples were washed in an ascending concentration of ethanol and propylene

oxide and embedded in Epon 812. Thin sections were doubly stained with uranyl acetate and lead citrate and examined under a Hitachi H-7650 transmission electron microscope.

Human and murine tissues and total RNA samples

The following human specimens were obtained from DV Biologics (Canada), Agilent Technologies (United States), and Biochain (United States); total RNA of fetal lung (17, 18, and 22 weeks gestation, Agilent Technologies, #540177, Lot.0006055802), that of adult lung (40 years of age, Agilent Technologies, #540019, Lot.0006118369), that of fetal thyroid (36 weeks gestation, Biochain, #R1244265-10, Lot.A811069), that of adult thyroid (38, 60, and 68 years of age, Life technologies, #AM6000, Lot.1102082), that of fetal throat (36 weeks gestation, Biochain, # R1244263-10, Lot.B212133), that of fetal liver (20 weeks gestation, Biochain, # R1244149-50, Lot.A601605), that of postnatal thymus (6, 6, and 10 months of age, Life technologies, #AM6000, Lot.1102082), that of adult brain (59, 78, and 85 years of age, Life technologies, #AM6000, Lot.1102082), that of adult pancreas (83 years of age, Life technologies, #AM7954, Lot.0910004), frozen tissue of fetal lung (18.5 weeks gestation, DV Biologics, PP001-FS, Lot.102508RH), that of adult thyroid (25 years of age, Biochain, #T1234265-RT1, Lot.A709031), and fetal lung fibroblasts (17.5 weeks

gestation, DV Biologics, # PP002-F-1349, Lot.121109VA). All murine specimen were derived from C58BL/6 strain.

SUPPLEMENTAL REFERENCES

Nannya Y, Sanada M, Nakazaki K, Hosoya N, Wang L, Hangaishi A, Kurokawa M, Chiba S, Bailey DK, Kennedy GC, Ogawa S. (2005). A robust algorithm for copy number detection using highdensity oligonucleotide single nucleotide polymorphism genotyping arrays. Cancer Res. *65*, 6071–6079.

Osanai K, Higuchi J, Oikawa R, Kobayashi M, Tsuchihara K, Iguchi M, Huang J, Voelker DR, Toga H. (2010). Altered lung surfactant system in a Rab38-deficient rat model of Hermansky-Pudlak syndrome. Am. J. Physiol. Lung Cell Mol. Physiol. *298*, L243-251.

Schmeckebier S, Mauritz C, Katsirntaki K, Sgodda M, Puppe V, Duerr J, Schubert SC, Schmiedl A, Lin Q, Paleček J, Draeger G, Ochs M, Zenke M, Cantz T, Mall MA, Martin U. (2013). Keratinocyte growth factor and dexamethasone plus elevated cAMP levels synergistically support pluripotent stem cell differentiation into alveolar epithelial type II cells. Tissue Eng. Part A. *19*, 938-951.

Yamamoto G, Nannya Y, Kato M, Sanada M, Levine RL, Kawamata N, Hangaishi A,

Kurokawa M, Chiba S, Gilliland DG, Koeffler HP, Ogawa S. (2007). Highly sensitive method for genomewide detection of allelic composition in nonpaired, primary tumor specimens by use of affymetrix single-nucleotide- polymorphism genotyping microarrays. Am. J. Hum. Genet. *81*, 114–126.
