# Supplementary material for: Generation of Alveolar Epithelial Spheroids via Isolated Progenitor Cells from Human Pluripotent Stem Cells
Source: Stem Cell Reports. 2014 Aug 21;3(3):394–403. doi: 10.1016/j.stemcr.2014.07.005 (PMC4266003; doi:10.1016/j.stemcr.2014.07.005)
Supplement: Document S2. Article plus Supplemental Information [file mmc2.pdf]

Stem Cell Reports
Report

OPEN ACCESS

Generation of Alveolar Epithelial Spheroids via Isolated Progenitor Cells from Human Pluripotent Stem Cells

Shimpei Gotoh,[1,*] Isao Ito,[1,2,*] Tadao Nagasaki,[1] Yuki Yamamoto,[1] Satoshi Konishi,[1] Yohei Korogi,[1] Hisako Matsumoto,[1] Shigeo Muro,[1] Toyohiro Hirai,[1] Michinori Funato,[3] Shin-Ichi Mae,[3] Taro Toyoda,[3] Aiko Sato-Otsubo,[4] Seishi Ogawa,[4] Kenji Osafune,[3] and Michiaki Mishima[1]

[1]Department of Respiratory Medicine, Graduate School of Medicine
[2]Institute for Integrated Cell-Material Sciences (iCeMS)
[3]Center for iPS Cell Research and Application (CiRA)
[4]Department of Pathology and Tumor Biology, Graduate School of Medicine
Kyoto University, Kyoto 606-8507, Japan
*Correspondence: a0009650@kuhp.kyoto-u.ac.jp (S.G.), isaoito@kuhp.kyoto-u.ac.jp (I.I.)
http://dx.doi.org/10.1016/j.stemcr.2014.07.005
This is an open access article under the CC BY license (http://creativecommons.org/licenses/by/3.0/).

SUMMARY

No methods for isolating induced alveolar epithelial progenitor cells (AEPCs) from human embryonic stem cells (hESCs) and induced pluripotent stem cells (hiPSCs) have been reported. Based on a study of the stepwise induction of alveolar epithelial cells (AECs), we identified carboxypeptidase M (CPM) as a surface marker of NKX2-1^{+} "ventralized" anterior foregut endoderm cells (VAFECs) in vitro and in fetal human and murine lungs. Using *SFTPC-GFP* reporter hPSCs and a 3D coculture system with fetal human lung fibroblasts, we showed that CPM^{+} cells isolated from VAFECs differentiate into AECs, demonstrating that CPM is a marker of AEPCs. Moreover, 3D coculture differentiation of CPM^{+} cells formed spheroids with lamellar-body-like structures and an increased expression of surfactant proteins compared with 2D differentiation. Methods to induce and isolate AEPCs using CPM and consequently generate alveolar epithelial spheroids would aid human pulmonary disease modeling and regenerative medicine.

INTRODUCTION

Type II alveolar epithelial cells (AECs) are a major cellular component of the distal lung epithelium, where they secrete pulmonary surfactant and generate type I AECs that cover most of the surface area of the alveoli (Whitsett et al., 2010; Rock and Hogan, 2011). The stepwise differentiation of human pluripotent stem cells (hPSCs), including human embryonic stem cells (hESCs) and induced pluripotent stem cells (hiPSCs), into lung epithelial cells would help to elucidate the etiologies of human lung diseases and create novel treatments, and has been reported in both proximal airway cells (Mou et al., 2012; Wong et al., 2012; Firth et al., 2014) and distal lung epithelial cells (Green et al., 2011; Ghaedi et al., 2013; Huang et al., 2014). Currently, however, there are no surface markers that can be used to purify human NKX2-1^{+} "ventralized" anterior foregut endoderm cells (VAFECs) as alveolar epithelial progenitor cells (AEPCs), although NKX2-1 is an early marker of lung and thyroid development (Kimura et al., 1996). Here, we report the efficacy of carboxypeptidase M (CPM) as a surface marker of AEPCs for generating type II AECs.

RESULTS

Identification of CPM as a Marker of NKX2-1^{+} VAFECs

We hypothesized that identifying a surface marker for NKX2-1^{+} VAFECs would be helpful for isolating a homogeneous population of AEPCs without establishing *NKX2-1* reporter cell lines. We constructed a stepwise protocol to induce hPSCs to AECs (Figure 1A). On day 0, previously established hPSCs were seeded (Thomson et al., 1998; Takahashi et al., 2007; Nakagawa et al., 2008; Okita et al., 2013) following single-cell enzymatic dissociation (Kajiwara et al., 2012), resulting in definitive endodermal cells (DECs) at an efficiency of $\geq$80% (Figure S1A available online). In step 2, the DECs were differentiated to anterior foregut endodermal cells (AFECs) (Green et al., 2011) at an efficiency of $\geq$88% (Figure S1B). In step 3, the concentrations of all-*trans* retinoic acid, CHIR99021, and BMP4 were optimized for seven hPSC lines for differentiation into NKX2-1^{+}FOXA2^{+} cells, attaining an efficiency of 57.0%–77.5% (Figures 1C and 1D; Supplemental Experimental Procedures). In step 4, cells were cultured in medium containing FGF10 for 7 days. In step 5, the cells were differentiated in medium containing dexamethasone, 8-Br-cAMP, 3-isobutyl-1-methylxanthine, and KGF (Gonzales et al., 2002; Longmire et al., 2012). We confirmed induction of AECs by detecting *SFTPB* and *SFTPC* using RT-PCR and double staining SFTPC and SFTPB with NKX2-1 (Figures S1C and S1D). Transcription factors were analyzed by quantitative RT-PCR (qRT-PCR; Figure 1B). *SOX17*, *FOXA2*, *GATA6*, and *SOX2* were compatibly changed on day 6 and day 10 as previously described (Green et al., 2011). On day 14, *NKX2-1*, *GATA6*, *ID2*, *SOX9*, and *HOPX* levels

simultaneously increased. Interestingly, *NKX2-1*, *GATA6*, and *HOPX* levels decreased on day 21 and then increased again on day 25. The levels of other organ lineage markers were found to be limited from day 0 to day 25 (Figure S1E).

In order to identify candidate markers of VAFECs, we performed a microarray analysis to compare the global gene-expression patterns of AFECs (day 10) and VAFECs (day 14) in 201B7 hiPSCs. *CPM* and *NKX2-1* were remarkably upregulated on day 14 (Figures 1E and S1F). In immunofluorescence (IF) staining, CPM and NKX2-1 increased from day 10 to day 14 (Figure 1F), whereas EPCAM and FOXA2 did not appear to change (Figure S1G). Although CPM was reported to be a marker of type I AECs (Nagae et al., 1993), only *CPM* drastically increased on day 14 in a similar pattern to *NKX2-1*, in contrast to other markers of type I AECs (*AQP5* and *CAV1*) (Figure S1H). On day 25, the various airway markers, including distal lung transcription factors (SOX9 and NKX2-1), type II AEC markers (SFTPB and SFTPC), and a club cell marker (SCGB3A2), were expressed in the CPM$^+$ cells. KRT5, a marker of basal cells, was not expressed in the CPM$^+$ cells (Figure 1G).

In fetal human lung at 18.5 weeks of gestation, SFTPC and T1α were expressed separately (Figure S1I), while CPM was expressed in NKX2-1$^+$, SFTPC$^+$, and T1α^+ cells (Figure 1H), thus indicating that CPM is expressed in both type I and II AECs in the fetus. The sequential expression of CPM was confirmed in NKX2-1$^+$ cells of fetal murine lungs at embryonic day 12.5 (E12.5), E15.5, and E17.5 (Figure 1I). For thyroid lineage cells, which differentiated from NKX2-1$^+$ VAFECs, CPM was found to be negative in both NKX2-1$^+$ cells and PAX8$^+$ epithelial cells, but weakly positive in PECAM$^+$ endothelial cells in the adult human samples (Figure S1J). In E17.5 fetal and adult murine thyroids, CPM was also negative in NKX2-1$^+$ and PAX8$^+$ cells (Figure S1K), suggesting that CPM is a lung-lineage marker of VAFECs.

Isolation of NKX2-1$^+$ VAFECs Using Anti-CPM$^+$ Antibody

As CPM is a membrane-bound surface protein, we performed flow cytometry with anti-EPCAM and anti-CPM antibodies after dissociating VAFECs on day 14 (Figures 2A and S2A). We then sorted EPCAM$^+$CPM$^+$ and EPCAM$^+$CPM$^-$ cells and examined the global gene-expression patterns of these two populations using microarrays. We screened 560 probes with a false discovery rate (FDR)-adjusted p value of <0.05 among 54,675 probes. Gene clustering was performed in 336 probes that differed between the EPCAM$^+$CPM$^+$ and EPCAM$^+$CPM$^-$ cells with a fold change (FC) cutoff value of 2.0 (Figures 2B and S2B). Of the clustered genes with the highest expression in the EPCAM$^+$CPM$^+$ cells, *CPM* ranked among the top five probes with a log FC of >6, as expected. Importantly, the log FCs of two probes for *NKX2-1* were 4.89 and 4.82, respectively. *FOXA1*, *FOXA2*, *HOPX*, and *GATA6* were also included in the list of upregulated genes with log FCs of 3.79, 3.06, 3.61, and 3.29, respectively. Next we sorted the CPM$^+$ cells using a magnet-activated cell sorting (MACS) system to increase the yield, as almost all of the CPM$^+$ cells were EPCAM$^+$ cells (96.7% ± 2.1% of CPM$^+$ cells; Figure 2A). After MACS-based sorting, the proportion of CPM$^+$ cells in three populations (presorting, positive selection, and negative selection) was 63.4% ± 5.8%, 98.8% ± 0.4%, and 34.0% ± 7.8%, respectively, by flow cytometry (Figure 2C). We then evaluated the proportion of positive NKX2-1$^+$ cells among the MACS-sorted CPM$^+$ and CPM$^-$ cells using IF staining (93.0% ± 1.0% versus 29.0% ± 1.0%; Figure S2C) and flow cytometry (92.3% ± 0.7% versus 22.2% ± 2.3%; Figure S2D). Because a portion of the CPM$^+$ cells appeared to be sorted according to MACS-based CPM negative selection, we investigated the average proportion of NKX2-1$^+$ cells among the fluorescence-activated cell sorting (FACS)-sorted CPM$^+$ and CPM$^-$ cells using IF staining (89.9% ± 0.4% versus 4.5% ± 1.7%; Figure 2D). Following CPM-based purification on day 14, *CPM* increased significantly from 0.74-fold ± 0.12-fold to 4.94-fold ± 0.51-fold of that observed in the fetal human lung (n = 5), while *NKX2-1* increased from 0.41-fold ± 0.10-fold to 1.95-fold ± 0.36-fold (n = 5) on qRT-PCR (Figure 2E). We then applied this method to purify AECs on day 25. *CPM, NKX2-1, SFTPA2*, *SFTPB*, *SFTPC*, *DCLAMP*, *SCGB1A1*, and *SCGB3A2* were significantly increased in the CPM$^+$ cells (n = 5); however, the level of *SFTPC* was extremely low compared with that observed in the fetal lung. *NGFR*, a marker of proximal airway basal stem cells (Rock et al., 2009), was significantly decreased in the CPM$^+$ cells (n = 5; Figure 2F).

Generation of *SFTPC-GFP* Knockin Reporter hPSCs

In order to investigate whether CPM is a potential surface marker of AEPCs, we generated *SFTPC-GFP* knockin reporter hPSC lines from 201B7 hiPSCs using BAC-based homologous recombination methods (Mae et al., 2013; Figure 3A; Supplemental Experimental Procedures), as SFTPC is the most specific marker of type II AECs. Following electroporation of the targeting vectors, 12 of 55 G418-resistant clones were found to have a heterozygous deletion of the genomic endogenous *SFTPC*-coding region (Figure 3B). The *pgk-Neo* cassette was removed via electroporation of the Cre-expression vector (Figure 3C), and normal karyotypes of the A17-14 and B2-3 clones were confirmed (Figure S3). The genomic copy number was calculated as previously described (Mae et al., 2013). The parental 201B7 (data not shown), A17-14, and B2-3 clones have

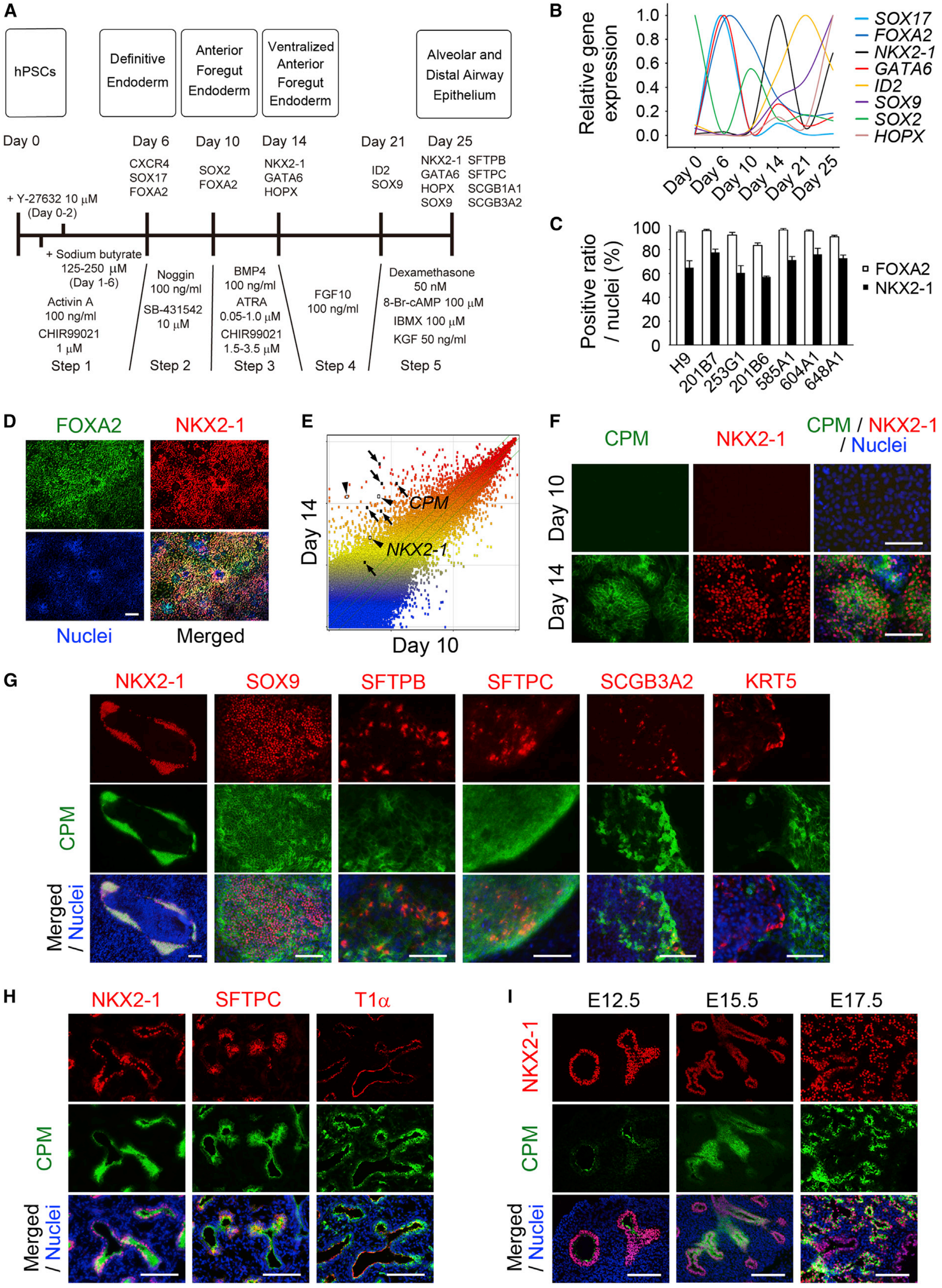

(legend on next page)

two copies of the *SFTPC* gene loci, in contrast to the A17-13 clone, in which random transgenic integration is supposed to have occurred, as indicated by three copies of the loci (Figure 3D). No copy-number variation was detected for the B2-3 clone, whereas a copy-number loss at chromosome 16 q23.3 and gain at chromosome 20 p13 were detected for the A17-14 clone (data not shown). Both *SFTPC-GFP* reporter hPSCs were then differentiated to the end of step 5 and GFP$^+$ and GFP$^-$ cells were obtained by FACS after the CPM$^+$ cells were sorted using MACS (Figure 3E). We confirmed the correlation between *GFP* and *SFTPC* on RT-PCR (Figure 3F). GFP was detected in SFTPC$^+$, SFTPB$^+$, and NKX2-1$^+$ cells for both clones (Figure 3G).

Alveolar Differentiation from CPM$^+$ VAFECs in 3D Coculture

We attempted 2D differentiation, reseeding the CPM$^+$ *SFTPC-GFP* reporter hPSCs purified from VAFECs on day 14 onto Matrigel-coated, 96-well plates. After 14 days of differentiation in step 5 medium, SFTPB became positive in the reseeded CPM$^+$ cells (Figure S2E); however, *SFTPC* was almost negative (Figure S4D, condition b). We obtained similar results when we sorted and reseeded CPM$^+$ cells on day 23 (Figure S2F). The discrepancy between the expression of SFTPB and SFTPC in developing human lungs was previously reported (Khoor et al., 1994). Therefore, we hypothesized that some missing factors are important for the coexpression of SFTPB and SFTPC. We then adopted a 3D coculture with fetal human lung fibroblasts (FHLFs) obtained at 17.5 weeks of gestation (Figure 4A). CPM$^+$ cells purified from VAFECs on day 14 and FHLFs were mixed at a ratio of 1:50 and reseeded onto cell inserts. After 10 days of differentiation in step 5 medium, GFP became positive in some spheroids (Figure 4B). The spheroids were subsequently examined with a transmission electron microscope and lamellar-body-like structures were noted (Figure 4C). On hematoxylin-and-eosin staining, cyst-like spheroids consisting of pseudostratified, columnar, or cuboidal cells with dark pink cytoplasm were observed in the CPM$^+$ cell-derived spheroids, whereas small pieces of spheroids consisting of cuboidal cells with clear cytoplasm were noted in the CPM$^-$ cell-derived spheroids (Figure S4A). On IF staining, CPM and NKX2-1 were double positive in most CPM$^+$ cell-derived spheroids, while GFP and SFTPC were double positive in some spheroids (Figure 4D). In the CPM$^-$ cell-derived spheroids, EPCAM was positive, whereas no CPM$^+$ or NKX2-1$^+$ cells were identified (Figure S4B). SFTPA, SFTPB, SFTPC, and SFTPD (representative markers of type II AECs) were positive in the CPM$^+$ cell-derived spheroids (Figure S4C). AQP5$^+$ cells were adjacent to SFTPC$^+$ cells in some spheroids (Figure 4D). ID2 and SOX9 (markers of differentiation into the distal lung-lineage fate) were positive in some NKX2-1$^+$ and CPM$^+$ cells, respectively (Figure S4C). Next, we trypsinized the cells in 3D structures and determined the proportion of SFTPC-GFP$^+$ cells, detecting 3.82% ± 0.50% cells obtained from the CPM$^+$ cell-derived 3D structures and 0.29% ± 0.03% cells obtained from the CPM$^-$ cell-derived structures including fibroblasts (Figure 4E). Excluding the fibroblasts, the ratio of the number of SFTPC-GFP$^+$ cells to that of EPCAM$^+$ cells was calculated to be 9.81% ± 1.81% in the CPM$^+$ cell-derived spheroids and 1.07% ± 0.16% in the CPM$^-$ cell-derived spheroids. Almost all of the GFP$^+$ cells sorted by FACS were SFTPC$^+$, whereas the GFP$^-$ cells were SFTPC$^-$ (Figure 4F). The levels of alveolar markers (*SFTPB* and *SFTPC*), rather than club cell markers (*SCGB1A1* and *SCGB3A2*), were significantly elevated following the 3D coculture differentiation of CPM$^+$ cells derived from three hPSC lines (H9 hESCs and parental 201B7 and 604A1 hiPSCs) compared with the 2D differentiation employing the three protocols separately starting on day 14 (Figure 1A; Green et al., 2011; Longmire et al., 2012) and the 3D coculture differentiation of CPM$^-$ cells (Figure 4G). Interestingly, the levels of *SFTPB* and *SFTPC* were quite low for 585A1 hiPSCs, suggesting

Figure 1. Identification of CPM as a Candidate Marker of NKX2-1$^+$ VAFECs
(A) Stepwise differentiation to AECs from hPSCs.
(B) Gene-expression levels of transcription factors from day 0 to day 25 (n = 3). Each value was normalized to the level of *β-ACTIN*. The relative expression level was scored with the maximum value set to 1.0.
(C) Induction efficiency of VAFECs analyzed by scoring the number of FOXA2$^+$ and NKX2-1$^+$ cells relative to the total number of nuclei in an average of ten randomly selected images (n = 3).
(D) FOXA2$^+$NKX2-1$^+$ VAFECs derived from 201B7 hiPSCs.
(E) Scatterplots comparing the global gene-expression profiles of AFECs (day 10) and VAFECs (day 14). *CPM* (arrows) and *NKX2-1* (arrowheads) are noted. The lines beside the diagonal line indicate a 2-fold cutoff change between the AFECs and VAFECs.
(F) Simultaneous increases of CPM and NKX2-1 detected by IF staining of AFECs (day 10) and VAFECs (day 14).
(G) CPM detected in NKX2-1$^+$, SOX9$^+$, SFTPB$^+$, SFTPC$^+$, and SCGB3A2$^+$ cells, but not in KRT5$^+$ cells, on day 25.
(H) CPM detected in NKX2-1$^+$ lung epithelial cells in fetal human lung.
(I) CPM in E12.5, E15.5, and E17.5 murine lungs.
Error bars show SEM. Scale bars, 100 μm. See also Figure S1 and Tables S1 and S2.

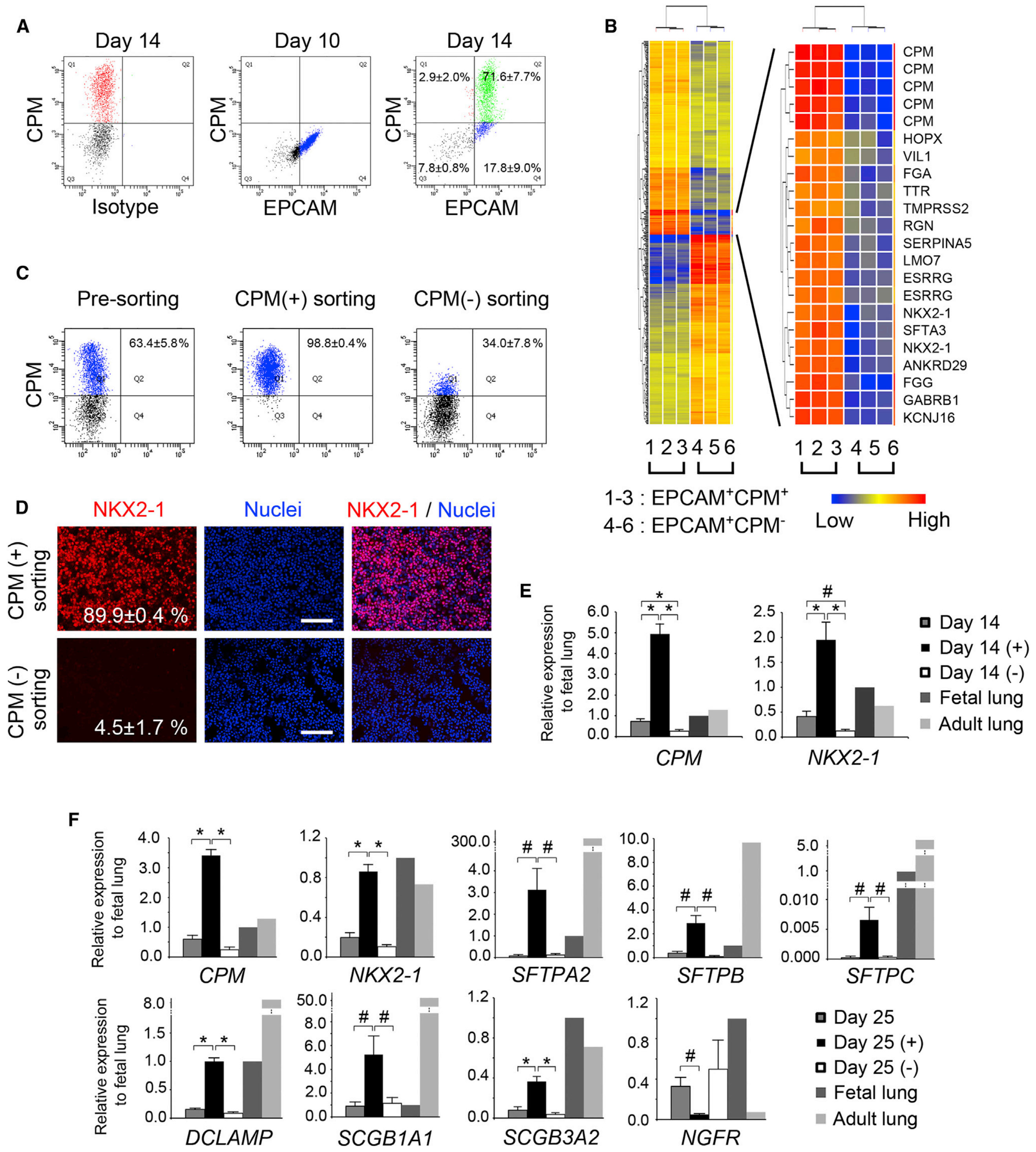

Figure 2. Isolation of CPM$^+$ VAFECs Using Anti-CPM Antibody

(A) Flow cytometry of VAFECs. EPCAM$^+$CPM$^+$ (Q2) and EPCAM$^+$CPM$^-$ cells (Q4) were isolated on day 14 (n = 3).

(B) Hierarchical clustering heatmaps of 336 genes with differences of >2-fold (FDR-adjusted $p < 0.05$) comparing EPCAM$^+$CPM$^+$ cells with EPCAM$^+$CPM$^-$ cells. The cluster of genes increased as the greatest fold change was magnified.

(C) Flow cytometry of MACS-sorted CPM$^+$ and CPM$^-$ cells from VAFECs (n = 3).

(D) NKX2-1$^+$ cells in FACS-sorted CPM$^+$ and CPM$^-$ cells derived from VAFECs analyzed by scoring the number of NKX2-1$^+$ cells relative to the total number of nuclei in an average of five randomly selected images (n = 3).

(legend continued on next page)

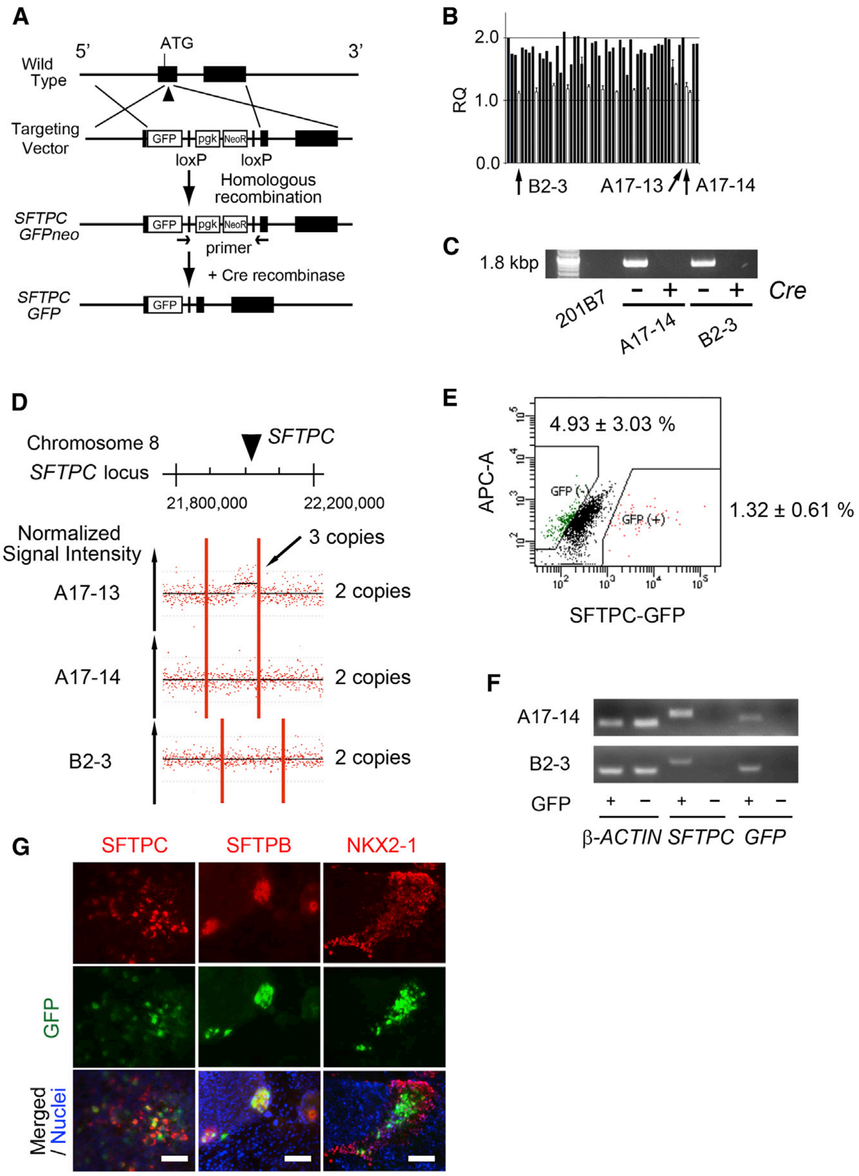

Figure 3. Generation of *SFTPC-GFP* Knockin hPSC Lines

(A) Strategy for BAC-based gene targeting to produce *SFTPC-GFP* knockin hPSC lines.

(B) Screening of knockin hPSC lines using TaqMan qPCR. Positive clones with candidate heterozygous deletion of the endogenous *SFTPC* gene are shown in white bars. Only clones that were initially suspected to be positive were tested independently three times.

(C) Genomic PCR to confirm the removal of the *pgk-NeoR* cassette by Cre-recombinase in the A17-14 and B2-3 *SFTPC-GFP* reporter hPSCs.

(D) SNP array analysis of the *SFTPC-GFP* knockin hPSC lines. The copy number of *SFTPC* gene loci was analyzed in A17-13, A17-14, and B2-3 clones. The A17-14 and B2-3 clones have two copies of the *SFTPC* gene loci, whereas the A17-13 clone has three copies of the loci. The red dots and y axis represent the normalized signal intensity of each SNP.

(E) Isolation of SFTPC$^-$GFP$^+$ and GFP$^-$ cells via FACS after sorting CPM$^+$ cells via MACS on day 25.

(F) RT-PCR analyses of GFP$^+$ and GFP$^-$ sorted cells in the A17-14 and B2-3 *SFTPC-GFP* reporter hPSC lines.

(G) Representative images of GFP detected in SFTPC$^+$, SFTPB$^+$, and NKX2-1$^+$ cells.

Error bars show SEM. Scale bars, 100 μm. See also Figure S3 and Tables S1 and S2.

that the concentration of retinoic acid required to induce NKX2-1$^+$ VAFECs in step 3 is less important for subsequent differentiation into AECs than the difference in the cell lines or donors. Moreover, the expression of *SFTPB* and *SFTPC* was small for the 2D and 3D differentiation of CPM$^+$ cells alone or FHLFs alone (Figure S4D). Finally, other cell-type markers (*AQP5* [type I AECs], *FOXJ1* [ciliated cells], and *AGR2* [goblet cells]) appeared to be elevated in the CPM$^+$ cell-derived structures rather than in the CPM$^-$ cell-derived structures, suggesting that cell-type markers other than club-cell markers were expressed in the CPM$^+$ cell-derived spheroids. *KRT5* (a basal cell marker, possibly including both airway and esophageal basal cells) was exclusively expressed in the CPM$^-$

(E) Levels of *CPM* and *NKX2-1* on day 14 before and after MACS-based purification of CPM$^+$ cells on qRT-PCR (n = 5).

(F) Levels of AEC and club-cell markers and *NGFR*, a proximal airway stem cell marker, on day 25 before and after MACS-based purification of CPM$^+$ cells (n = 5).

The gene-expression level observed in the fetal lungs was set at one. Values are presented as the mean ± SEM. Error bars show SEM. #p < 0.05, *p < 0.01. Scale bars, 100 μm. See also Figure S2 and Tables S1 and S2.

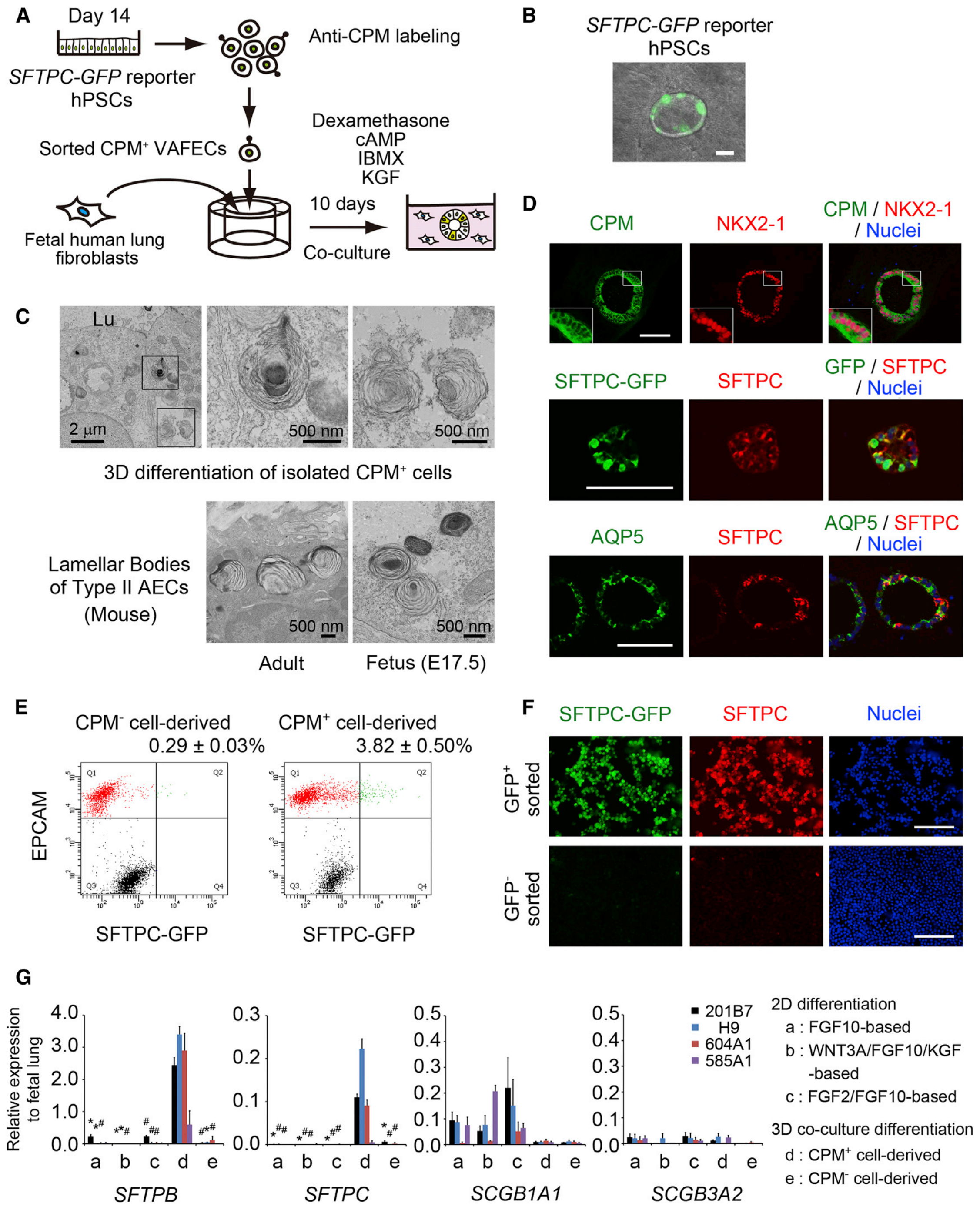

Figure 4. Alveolar Differentiation from CPM$^+$ VAFECs in 3D Coculture

(A) Strategy for inducing AECs via 3D coculture with FHLFs.

(B) SFTPC-GFP$^+$ cells detected in spheroids derived from isolated CPM$^+$ VAFECs.

(C) Transmission electron microscopy of lamellar-body-like structures observed in 3D coculture differentiation of CPM$^+$ cells compared with those observed in the adult and fetal murine lungs. Lu, lumen.

(D) IF staining of spheroids derived from CPM$^+$ VAFECs.

(E) Flow cytometry of SFTPC-GFP$^+$ cells in 3D coculture differentiation of CPM$^+$ cells or CPM$^-$ cells (n = 3).

(F) GFP$^+$ and GFP$^-$ cells isolated via FACS, spun down onto slides, and stained by anti-GFP and anti-SFTPC antibodies.

(legend continued on next page)

cell-derived structures. In addition, *PAX8* (a thyroid marker), *PAX6* (a neuron marker), and the other foregut endodermal lineage cells (*FOXN1*, *ALB*, and *PDX1*) were only minimally or slightly induced following 3D coculture differentiation (Figure S4E).

DISCUSSION

In this work, we identified CPM as a surface marker that is expressed in NKX2-1$^+$ VAFECs, including AEPCs, and demonstrated that the CPM$^+$ cell-derived spheroids obtained via 3D coculture differentiation with FHLFs enabled more efficient differentiation to AECs than did 2D differentiation. The gene-expression pattern of *CPM* in developing lungs has not received significant attention, although in situ hybridization of *Cpm* in anterior DECs as early as E7.5 in mice has been reported (Tamplin et al., 2008). Our data from IF staining of murine fetal lungs (Figure S1I) also suggest that lineage-tracing studies may provide answers to the following questions: Is Cpm a possible "specific" marker of lung-lineage progenitor cells such as Shh (Harris et al., 2006), Id2 (Rawlins et al., 2009a), and Nkx2-1 (Longmire et al., 2012)? What is the relationship between CPM$^+$ cells and bipotent cells that are capable of generating type I and type II AECs (Desai et al., 2014)? Do CPM$^+$ cells differentiate into type II AECs directly or indirectly via SFTPC$^+$SCGB1A1$^+$ cells (Kim et al., 2005; Rawlins et al., 2009b)? Furthermore, the present study suggests that a 3D microenvironment and coculture with FHLFs are important factors in the differentiation of progenitor cells into AECs rather than club cells. Although maintaining type II AECs in 2D conditions is often difficult (Dobbs, 1990; Yu et al., 2007), 3D conditions have recently been applied with better outcomes (Yu et al., 2007; McQualter et al., 2010; Barkauskas et al., 2013). Therefore, our 3D differentiation protocol appears to be a reasonable approach for maintaining differentiated type II AECs, although methods for expanding such cells for longer periods should be established in the next step.

The limitations of the present study include the fact that we were unable to demonstrate whether CPM is a more appropriate marker for lung-lineage cells than NKX2-1. Future studies focusing on the possible contribution of NKX2-1$^-$CPM$^+$ cells and/or NKX2-1$^+$CPM$^-$ cells to the differentiation of lung epithelial cells may resolve this issue, although we found only two isolatable populations of NKX2-1$^+$CPM$^+$ and NKX2-1$^-$CPM$^-$ cells using the present protocol. In addition, we were unable to demonstrate the highest induction efficiency of AECs, as recently described (Ghaedi et al., 2013), although we employed a different method for evaluating efficiency using *SFTPC-GFP* reporter hPSCs. Another limitation is that the functions of the induced AECs remain to be elucidated.

Nevertheless, the methods applied in the present study to induce and isolate AEPCs using CPM and consequently generate alveolar epithelial spheroids in a stepwise fashion may help to elucidate the complicated differentiation of human AECs and open the door for the development of new strategies for in vitro toxicology and cell replacement therapy, as well as screening for therapeutic drug compounds, in the future.

EXPERIMENTAL PROCEDURES

2D Differentiation

CHIR99021 (Axon Medchem), an activator of canonical Wnt signaling, was substituted for WNT3A (Mae et al., 2013). For details regarding the protocols used for each differentiation medium, see the Supplemental Experimental Procedures.

3D Differentiation

The protocol for the 3D culture was modified from a previous report (Barkauskas et al., 2013). For further details, see the Supplemental Experimental Procedures.

Ethics

The use of H9 hESCs was approved by the Ministry of Education, Culture, Sports, Science and Technology (MEXT) of Japan. Human ethics approval was obtained from the Institutional Review Board and Ethics Committee of Kyoto University Graduate School and Faculty of Medicine. Animal ethics approval was obtained from the Animal Ethics and Research Committee of Kyoto University.

Statistical Analysis

Values are expressed as the mean ± SEM and "n" stands for the number of independent experiments. Two-tailed Student's t test was performed to identify significant differences between two conditions of qRT-PCR.

ACCESSION NUMBERS

The NCBI GEO accession number for the microarray data reported in this paper is GSE53513.

(G) qRT-PCR comparing the 2D and 3D differentiation into AECs in H9 hESCs and 201B7 (parental), 604A1, and 585A1 hiPSCs. Each value of the gene expression was normalized to the level of *β-ACTIN*. The levels of the fetal lungs were set at one.
Values are presented as the mean ± SEM. Error bars show SEM. #$p < 0.05$, *$p < 0.01$. Scale bars, 100 μm unless otherwise indicated. See also Figure S4 and Tables S1 and S2.

SUPPLEMENTAL INFORMATION

Supplemental Information includes Supplemental Experimental Procedures, four figures, and two tables and can be found with this article online at http://dx.doi.org/10.1016/j.stemcr.2014.07.005.

AUTHOR CONTRIBUTIONS

S.G., I.I., and K.O. designed the study. S.G., T.N., Y.Y., S.K., Y.K., and A.S.-O. performed the experiments. S.G., I.I., T.N., Y.Y., S.K., A.S.-O., S.O., and K.O. analyzed the data. S.G. and I.I. wrote the manuscript through fruitful discussions with and supervision by H.M., S.M., T.H., S.O., K.O., and M.M. M.F., S.-I.M., T.T., and K.O. provided the method for inducing definitive endoderm and advised on the methods used for vector construction and other basic techniques.

ACKNOWLEDGMENTS

We are grateful to K. Okita, K. Takahashi, T. Aoi, M. Kajiwara, I. Asaka, and S. Yamanaka (Center for iPS Cell Research and Application, Kyoto University) for providing cell lines, plasmids, the method of iPSC culture, and endoderm differentiation. We thank K. Okamoto-Furuta and H. Kohda (Division of Electron Microscopic Study, Center for Anatomical Studies, Kyoto University) for technical assistance with the electron microscope. We also thank S. Tsukita and A. Tamura (Osaka University) for advice on electron microscopy, S. Kimura (National Cancer Institute, NIH) for providing the anti-SCGB3A2 antibody, Y. Ito for excellent advice and assistance, and Y. Maeda and A. Inazumi for technical assistance. Fluorescence studies and gene-expression microarray analyses were performed in part at the Medical Research Support Center of Kyoto University. This work was supported by Grants-in-Aid for Scientific Research (KAKENHI 22249031 and 23591146) from MEXT of Japan.

Received: January 17, 2014
Revised: July 18, 2014
Accepted: July 18, 2014
Published: August 21, 2014

REFERENCES

Barkauskas, C.E., Cronce, M.J., Rackley, C.R., Bowie, E.J., Keene, D.R., Stripp, B.R., Randell, S.H., Noble, P.W., and Hogan, B.L. (2013). Type 2 alveolar cells are stem cells in adult lung. J. Clin. Invest. *123*, 3025–3036.

Desai, T.J., Brownfield, D.G., and Krasnow, M.A. (2014). Alveolar progenitor and stem cells in lung development, renewal and cancer. Nature *507*, 190–194.

Dobbs, L.G. (1990). Isolation and culture of alveolar type II cells. Am. J. Physiol. *258*, L134–L147.

Firth, A.L., Dargitz, C.T., Qualls, S.J., Menon, T., Wright, R., Singer, O., Gage, F.H., Khanna, A., and Verma, I.M. (2014). Generation of multiciliated cells in functional airway epithelia from human induced pluripotent stem cells. Proc. Natl. Acad. Sci. USA *111*, E1723–E1730.

Ghaedi, M., Calle, E.A., Mendez, J.J., Gard, A.L., Balestrini, J., Booth, A., Bove, P.F., Gui, L., White, E.S., and Niklason, L.E. (2013). Human iPS cell-derived alveolar epithelium repopulates lung extracellular matrix. J. Clin. Invest. *123*, 4950–4962.

Gonzales, L.W., Guttentag, S.H., Wade, K.C., Postle, A.D., and Ballard, P.L. (2002). Differentiation of human pulmonary type II cells in vitro by glucocorticoid plus cAMP. Am. J. Physiol. Lung Cell. Mol. Physiol. *283*, L940–L951.

Green, M.D., Chen, A., Nostro, M.C., d'Souza, S.L., Schaniel, C., Lemischka, I.R., Gouon-Evans, V., Keller, G., and Snoeck, H.W. (2011). Generation of anterior foregut endoderm from human embryonic and induced pluripotent stem cells. Nat. Biotechnol. *29*, 267–272.

Harris, K.S., Zhang, Z., McManus, M.T., Harfe, B.D., and Sun, X. (2006). Dicer function is essential for lung epithelium morphogenesis. Proc. Natl. Acad. Sci. USA *103*, 2208–2213.

Huang, S.X., Islam, M.N., O'Neill, J., Hu, Z., Yang, Y.G., Chen, Y.W., Mumau, M., Green, M.D., Vunjak-Novakovic, G., Bhattacharya, J., and Snoeck, H.W. (2014). Efficient generation of lung and airway epithelial cells from human pluripotent stem cells. Nat. Biotechnol. *32*, 84–91.

Kajiwara, M., Aoi, T., Okita, K., Takahashi, R., Inoue, H., Takayama, N., Endo, H., Eto, K., Toguchida, J., Uemoto, S., and Yamanaka, S. (2012). Donor-dependent variations in hepatic differentiation from human-induced pluripotent stem cells. Proc. Natl. Acad. Sci. USA *109*, 12538–12543.

Khoor, A., Stahlman, M.T., Gray, M.E., and Whitsett, J.A. (1994). Temporal-spatial distribution of SP-B and SP-C proteins and mRNAs in developing respiratory epithelium of human lung. J. Histochem. Cytochem. *42*, 1187–1199.

Kim, C.F., Jackson, E.L., Woolfenden, A.E., Lawrence, S., Babar, I., Vogel, S., Crowley, D., Bronson, R.T., and Jacks, T. (2005). Identification of bronchioalveolar stem cells in normal lung and lung cancer. Cell *121*, 823–835.

Kimura, S., Hara, Y., Pineau, T., Fernandez-Salguero, P., Fox, C.H., Ward, J.M., and Gonzalez, F.J. (1996). The T/ebp null mouse: thyroid-specific enhancer-binding protein is essential for the organogenesis of the thyroid, lung, ventral forebrain, and pituitary. Genes Dev. *10*, 60–69.

Longmire, T.A., Ikonomou, L., Hawkins, F., Christodoulou, C., Cao, Y., Jean, J.C., Kwok, L.W., Mou, H., Rajagopal, J., Shen, S.S., et al. (2012). Efficient derivation of purified lung and thyroid progenitors from embryonic stem cells. Cell Stem Cell *10*, 398–411.

Mae, S., Shono, A., Shiota, F., Yasuno, T., Kajiwara, M., Gotoda-Nishimura, N., Arai, S., Sato-Otubo, A., Toyoda, T., Takahashi, K., et al. (2013). Monitoring and robust induction of nephrogenic intermediate mesoderm from human pluripotent stem cells. Nat. Commun. *4*, 1367.

McQualter, J.L., Yuen, K., Williams, B., and Bertoncello, I. (2010). Evidence of an epithelial stem/progenitor cell hierarchy in the adult mouse lung. Proc. Natl. Acad. Sci. USA *107*, 1414–1419.

Mou, H., Zhao, R., Sherwood, R.I., Ahfeldt, T., Lapey, A., Sicillian, L., Izvolsky, K.I., Musunuru, K., Cowan, C., and Rajagopal, J. (2012). Generation of multipotent embryonic lung and airway

progenitors from mouse ESCs and patient-specific cystic fibrosis iPSCs. Cell Stem Cell *10*, 385–397.

Nagae, A., Abe, M., Becker, R.P., Deddish, P.A., Skidgel, R.A., and Erdös, E.G. (1993). High concentration of carboxypeptidase M in lungs: presence of the enzyme in alveolar type I cells. Am. J. Respir. Cell Mol. Biol. *9*, 221–229.

Nakagawa, M., Koyanagi, M., Tanabe, K., Takahashi, K., Ichisaka, T., Aoi, T., Okita, K., Mochiduki, Y., Takizawa, N., and Yamanaka, S. (2008). Generation of induced pluripotent stem cells without Myc from mouse and human fibroblasts. Nat. Biotechnol. *26*, 101–106.

Okita, K., Yamakawa, T., Matsumura, Y., Sato, Y., Amano, N., Watanabe, A., Goshima, N., and Yamanaka, S. (2013). An efficient nonviral method to generate integration-free human-induced pluripotent stem cells from cord blood and peripheral blood cells. Stem Cells *31*, 458–466.

Rawlins, E.L., Clark, C.P., Xue, Y., and Hogan, B.L. (2009a). The Id2+ distal tip lung epithelium contains individual multipotent embryonic progenitor cells. Development *136*, 3741–3745.

Rawlins, E.L., Okubo, T., Xue, Y., Brass, D.M., Auten, R.L., Hasegawa, H., Wang, F., and Hogan, B.L. (2009b). The role of Scgb1a1+ Clara cells in the long-term maintenance and repair of lung airway, but not alveolar, epithelium. Cell Stem Cell *4*, 525–534.

Rock, J.R., and Hogan, B.L. (2011). Epithelial progenitor cells in lung development, maintenance, repair, and disease. Annu. Rev. Cell Dev. Biol. *27*, 493–512.

Rock, J.R., Onaitis, M.W., Rawlins, E.L., Lu, Y., Clark, C.P., Xue, Y., Randell, S.H., and Hogan, B.L. (2009). Basal cells as stem cells of the mouse trachea and human airway epithelium. Proc. Natl. Acad. Sci. USA *106*, 12771–12775.

Takahashi, K., Tanabe, K., Ohnuki, M., Narita, M., Ichisaka, T., Tomoda, K., and Yamanaka, S. (2007). Induction of pluripotent stem cells from adult human fibroblasts by defined factors. Cell *131*, 861–872.

Tamplin, O.J., Kinzel, D., Cox, B.J., Bell, C.E., Rossant, J., and Lickert, H. (2008). Microarray analysis of Foxa2 mutant mouse embryos reveals novel gene expression and inductive roles for the gastrula organizer and its derivatives. BMC Genomics *9*, 511.

Thomson, J.A., Itskovitz-Eldor, J., Shapiro, S.S., Waknitz, M.A., Swiergiel, J.J., Marshall, V.S., and Jones, J.M. (1998). Embryonic stem cell lines derived from human blastocysts. Science *282*, 1145–1147.

Whitsett, J.A., Wert, S.E., and Weaver, T.E. (2010). Alveolar surfactant homeostasis and the pathogenesis of pulmonary disease. Annu. Rev. Med. *61*, 105–119.

Wong, A.P., Bear, C.E., Chin, S., Pasceri, P., Thompson, T.O., Huan, L.J., Ratjen, F., Ellis, J., and Rossant, J. (2012). Directed differentiation of human pluripotent stem cells into mature airway epithelia expressing functional CFTR protein. Nat. Biotechnol. *30*, 876–882.

Yu, W., Fang, X., Ewald, A., Wong, K., Hunt, C.A., Werb, Z., Matthay, M.A., and Mostov, K. (2007). Formation of cysts by alveolar type II cells in three-dimensional culture reveals a novel mechanism for epithelial morphogenesis. Mol. Biol. Cell *18*, 1693–1700.

Stem Cell Reports, Volume *3*

Supplemental Information

Generation of Alveolar Epithelial Spheroids via Isolated Progenitor Cells from Human Pluripotent Stem Cells

Shimpei Gotoh, Isao Ito, Tadao Nagasaki, Yuki Yamamoto, Satoshi Konishi, Yohei Korogi, Hisako Matsumoto, Shigeo Muro, Toyohiro Hirai, Michinori Funato, Shin-Ichi Mae, Taro Toyoda, Aiko Sato-Otsubo, Seishi Ogawa, Kenji Osafune, and Michiaki Mishima

Figure S1, related to Figure 1.

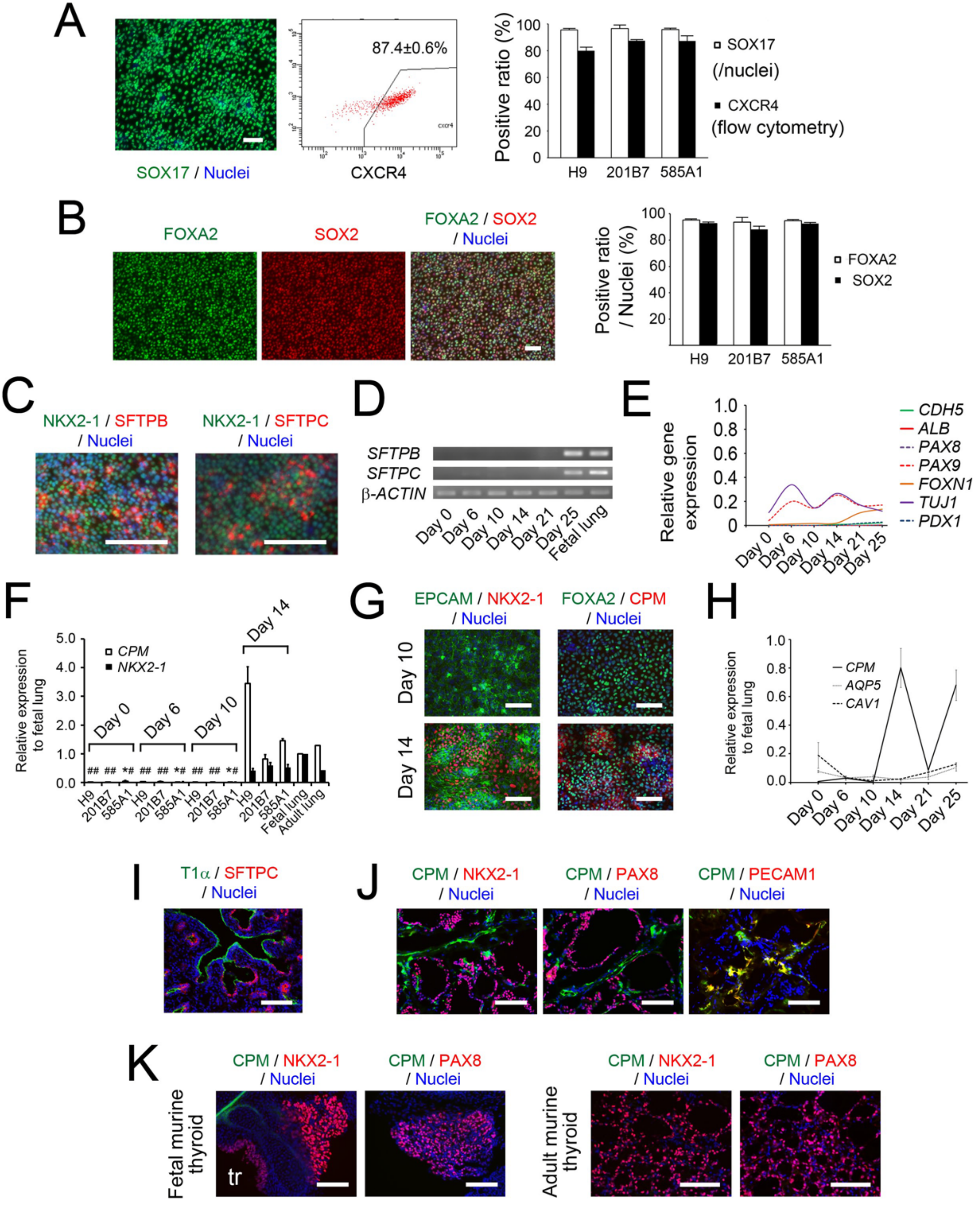

Figure S2, related to Figure 2.

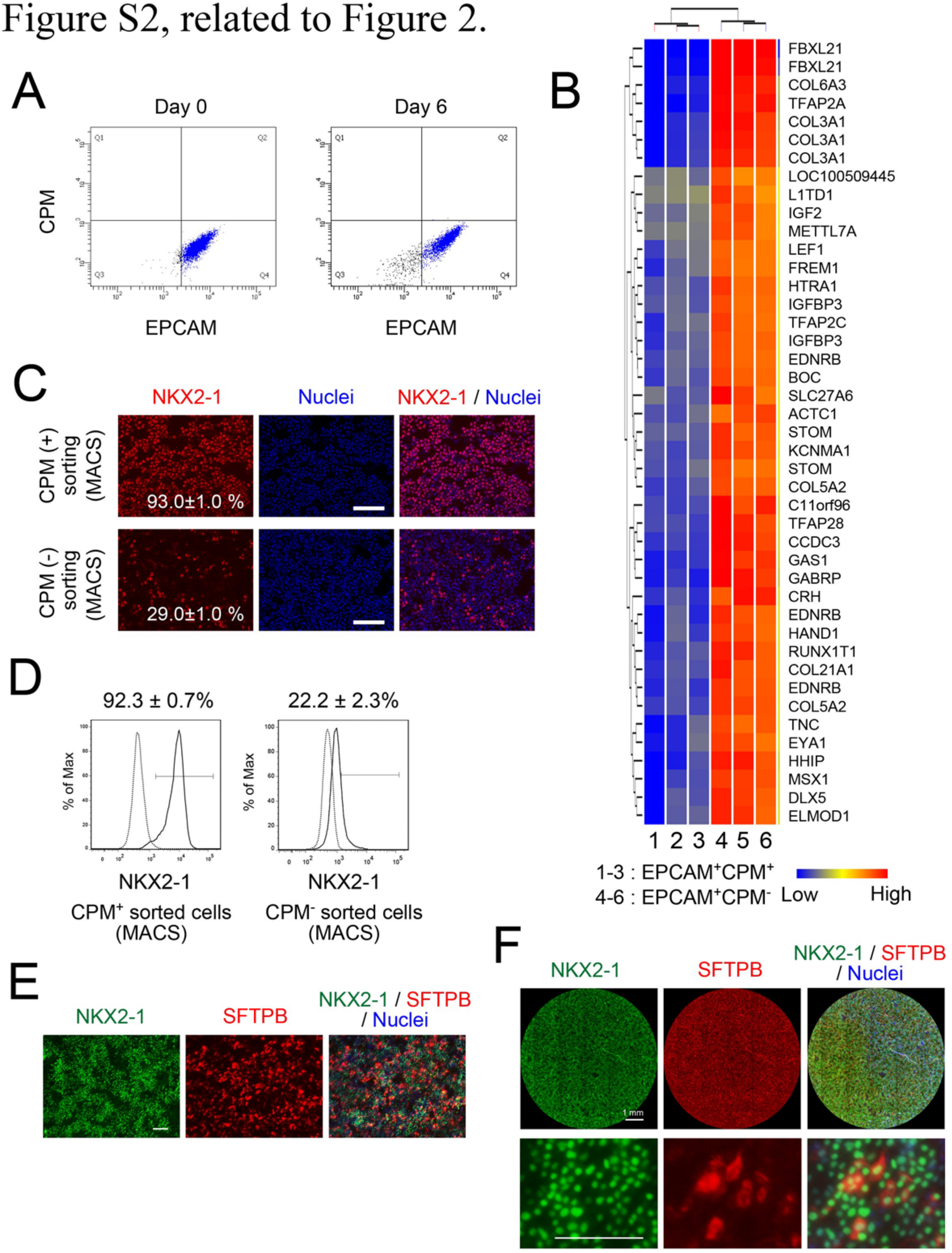

Figure S3, related to Figure 3.

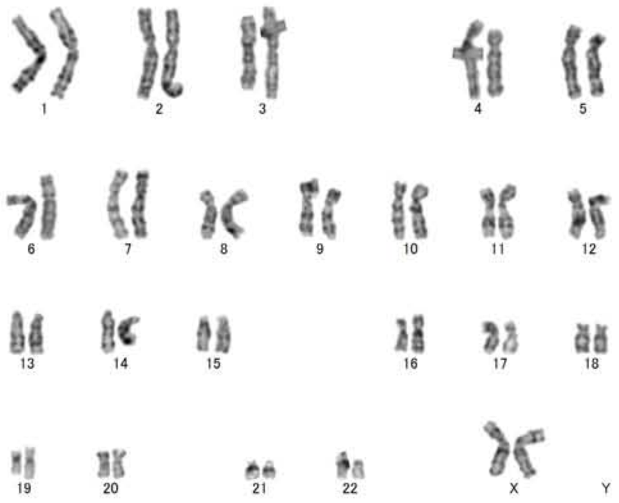

A17-14

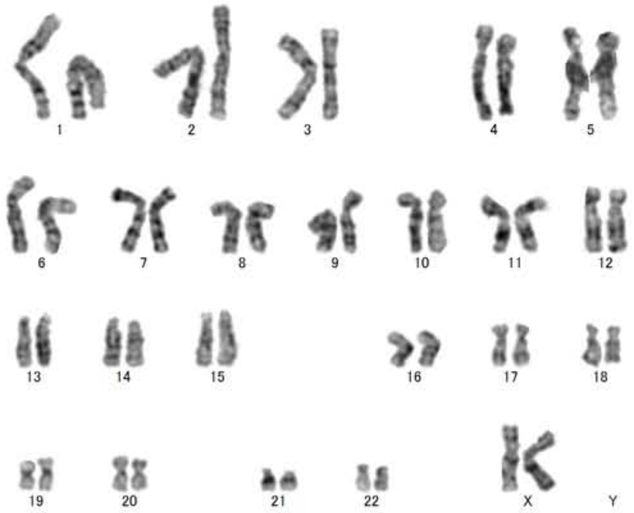

B2-3

Figure S4, related to Figure 4.

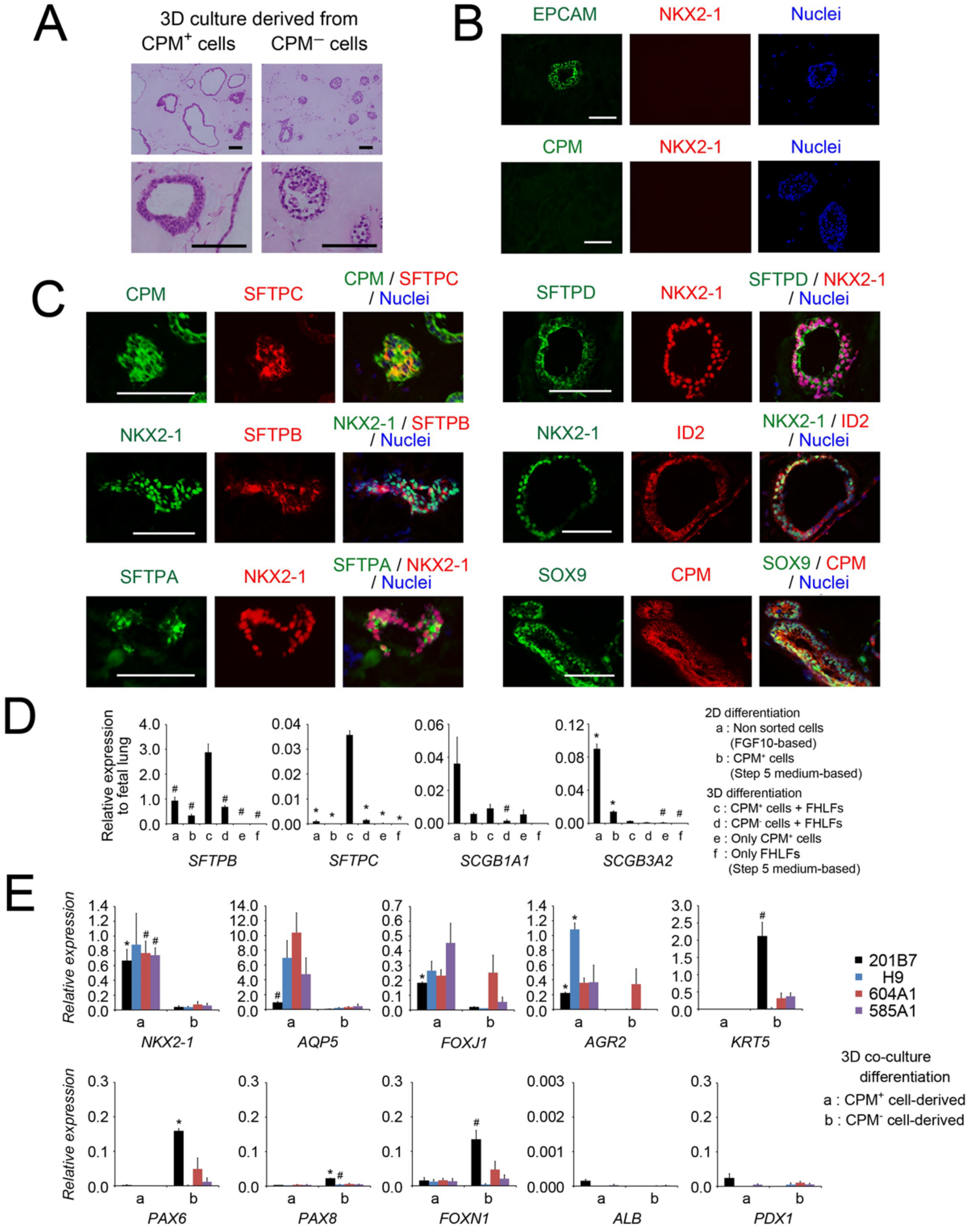

Table S1, related to Figure 1, 2, 3 and 4. Primers used in the present study.

Gene name		Primer Sequence	Size (bp)
β-ACTIN	F	CAATGTGGCCGAGGACTTTG	126
	R	CATTCTCCTTAGAGAGAAGTGG	
SOX17	F	CGCTTTCATGGTGTGGGCTAAGGACG	186
	R	TAGTTGGGGTGGTCCTGCATGTGCTG	
GATA6	F	CAGCAAAAATACTTCCCCCA	107
	R	ACTTGAGCTCGCTGTTCTCG	
FOXA2	F	TCGCTCTCCTTCAACGACTGTTTCC	107
	R	TTCTCGAACATGTTGCCCGAGTCAG	
SOX2	F	AGTCTCCAAGCGACGAAAAA	189
	R	TTTCACGTTTGCAACTGTCC	
NKX2-1	F	AGCACACGACTCCGTTCTC	68
	R	GCCCACTTTCTTGTAGCTTTCC	
CPM	F	TCCAAGGTGGAATGCAAGAT	181
	R	TCAAAAACTTGACCCTTTACACC	
SOX9	F	GAGGAAGTCGGTGAAGAACG	337
	R	ATCGAAGGTCTCGATGTTGG	
ID2	F	GACAGCAAAGCACTGTGTGG	144
	R	CCATTCAACTTGTCCTCCTTG	
HOPX	F	TCAACAAGGTCGACAAGCAC	157
	R	TCTGTGACGGATCTGCACTC	
SCGB1A1	F	TTCAGCGTGTCATCGAAACCC	189
	R	ACAGTGAGCTTTGGGCTATTTTT	
SCGB3A2	F	CAAGTGGAACCACTGGCTTG	198
	R	CCAGAGGTAAAGGTGCCAAC	
SFTPA2	F	AAGCAGCTGGAGGCTCTGT	88
	R	CCATCAAGATGAGGGTGAGG	
SFTPB	F	GAGCCGATGACCTATGCCAAG	133
	R	AGCAGCTTCAAGGGGAGGA	
SFTPC	F	GCAAAGAGGTCCTGATGGAG	178
	R	TGTTTCTGGCTCATGTGGAG	
DCLAMP	F	ACCGATGTCCAACTTCAAGC	161
	R	TGACACCTTAGGCGGATTTT	
AQP5	F	CTGTCCATTGGCCTGTCTGTC	248
	R	GGCTCATACGTGCCTTTGATG	
CAV1	F	AGGGCAACATCTACAAGCCC	188
	R	GCCGTCAAAACTGTGTGTCC	
NGFR	F	CGACAACCTCATCCCTGTCT	102
	R	TTGTTCTGCTTGCAGCTGTT	
KRT5	F	GAGCTGAGAAACATGCAGGA	82
	R	CAAGCGTACCACTGCTGAGA	
AGR2	F	AGCACTAGTGGGTGGGATTG	167
	R	GCAAGAATGCTGACACTGGA	
FOXJ1	F	CCTGTCGGCCATCTACAAGT	94
	R	AGACAGGTTGTGGCGGATT	
TUJ1	F	GCAACTACGTGGGCGACT	86
	R	TCGAGGCACGTACTTGTGAG	
PAX6	F	CGGAGTGAATCAGCTCGGTG	301
	R	CCGCTTATACTGGGCTATTTTGC	
PAX8	F	TCAACCTCCCTATGGACAGCTG	137
	R	GAGCCCATTGATGGAGTAGGTG	
PAX9	F	TGGTTATGTTGCTGGACATGGGTG	136
	R	GGAAGCCGTGACAGAATGACTACCT	
FOXN1	F	TGGAGAGTGGTGCTGGGATGTT	141
	R	GGTACTGATAGTGTGAGGAGCC	
CDH5	F	ACACCTCACTTCCCCATCA	95
	R	GACCTTGCCCACATATTCTCC	
PDX1	F	CCCATGGATGAAGTCTACC	262
	R	GTCCTCCTCCTTTTTCCAC	
ALB	F	CCTTTGGCACAATGAAGTGGGTAACC	164
	R	CAGCAGTCAGCCATTTCACCATAG	
EGFP	F	AGAACGGCATCAAGGTGAAC	135
	R	TGCTCAGGTAGTGGTTGTCG	

Table S2, related to Figure 1, 2, 3 and 4. Antibodies used in the present study.

Primary Antibodies	Dilution rate	Manufacturer	Clone / Cat. No.
GFP	1:500	Aves Labs	GFP-1020
SFTPA	1:100	Immuno-Biological Laboratories	PE10 / 10375
proSPB / SFTPB	1:2000	EMD-Millipore	AB3432
proSPC / SFTPC	1:2000	EMD-Millipore	AB3786
SFTPC	1:100	Santa Cruz Biotechnology	sc-13979
SFTPD	1:500	Yamasa corporation	10H11 /#7608
NKX2-1	1:500	Novus Biologocals	EP1584Y / NB100-80062
NKX2-1	1:500	Lab Vision	8G7G3/1 / MS-699-P
SCGB3A2	1:2000	a kind gift of Dr. Shioko Kimura	
CPM	1:500	Leica microsystems	1C2 / NCL-CPMm
CPM	1:250	Medical & Biological Laboratories	D293-3
AQP5	1:100	Santa Cruz Biotechnology	sc-9890
T1a / Podoplanin	1:100	Santa Cruz Biotechnology	18H5 / sc-59347
PECAM	1:100	Santa Cruz Biotechnology	sc-1506
EPCAM	1:100	Santa Cruz Biotechnology	EBA-1 / sc-66020
EPCAM (FITC)	1:10	BD Biosciences	EBA-1 / 347197
SOX17	1:500	R&D systems	AF1924
CXCR4 (PE)	1:10	R&D systems	12G5 / FAB170P
FOXA2	1:500	R&D systems	AF2400
SOX2	1:500	EMD-Millipore	AB5603
SOX9	1:20	R&D systems	AF3075
ID2	1:100	Santa Cruz Biotechnology	sc-489
KRT5	1:100	Lab Vision	EP1601Y / RM-2106-S0
PAX8	1:500	Proteintech	10336-1-AP
Mouse isotype control IgG1	1:100	Sigma-Aldrich	MOPC21 / M5284
Rabbit isotype control IgG	1:1000	Cell Signaling Technology	DA1E / #3900
Mouse isotype control IgG1 (FITC)	1:10	BD Biosciences	555748

Secondary Antibodies	Dilution rate	Manufacturer	Cat. No.
Donkey anti-mouse (Alexa546)	1:500	Life Technoloies	A10036
Donkey anti-mouse (Alexa488)	1:250	Life Technoloies	A21202
Goat anti-mouse (Alexa647)	1:200	Life Technoloies	A21236
Donkey anti-goat (Alexa488)	1:500	Life Technoloies	A11055
Donkey anti-goat (Cy3)	1:500	Jackson ImmunoResearch	705-165-147
Donkey anti-chicken (DyLight 488)	1:500	Jackson ImmunoResearch	703-485-155
Donkey anti-rabbit (Cy3)	1:500	Jackson ImmunoResearch	711-165-152
Donkey anti-rat (Alexa488)	1:500	Life Technoloies	A21208
Rat anti-mouse IgG1 (microbeads)	1:5	Miltenyi Biotec	130-047-101
Zenon Alexa488 Mouse IgG1 Labeling Kit	Manufacturer's protocol	Life Technoloies	Z-25002
Zenon Alexa555 Mouse IgG1 Labeling Kit	Manufacturer's protocol	Life Technoloies	Z-25005
Zenon Alexa647 Mouse IgG1 Labeling Kit	Manufacturer's protocol	Life Technoloies	Z-25008

SUPPLEMENTAL FIGURE LEGENDS

Figure S1. Related to Figure 1.

(A) Definitive endoderm cells (DECs) on Day 6. The efficiency of induction was examined by scoring the number of SOX17$^+$ cells relative to the total number of nuclei in an average of 10 randomly selected images (n=3) and counting the number of CXCR4$^+$ cells using flow cytometry. (B) Anterior foregut endoderm cells (AFECs) on Day 10. The efficiency was examined by scoring the number of FOXA2$^+$SOX2$^+$ cells relative to the total number of nuclei. (C) SFTPB and SFTPC detected in NKX2-1$^+$ cells on Day 25, respectively. (D) Electrophoresis of the RT-PCR products. Both *SFTPB* and *SFTPC* were positive only on Day 25 and in the positive control of the fetal human lungs. (E) RT-qPCR of *CDH5*, *ALB*, *PAX8*, *PAX9*, *FOXN1*, *TUJ1* and *PDX1*. Each gene expression level was normalized to that of *β-ACTIN* (n=3). The levels of positive controls in the fetal human lungs (*CDH5*), liver (*ALB*), thyroid (*PAX8*) and throat (*PAX9*), postnatal thymus (*FOXN1*) and the adult brain (*TUJ1*) and pancreas (*PDX1*) were set at 1. (F) RT-qPCR of *CPM* and *NKX2-1*. The levels of *CPM* and *NKX2-1* significantly increased from Day 0 to Day 14 in the H9, 201B7, and 585A1 cell lines (n=3). The gene expression observed in the fetal lungs was set at 1. (G) Expression of EPCAM and FOXA2 double-stained with NKX2-1 or CPM, respectively, on

Day 10 and Day 14. (H) Levels of *CPM* and other markers of type I AECs, including *AQP5* and *CAV1*, from Day 0 to Day 25 in the 201B7 cells (n=3). (I) T1α and SFTPC stained in the fetal human lung. (J) CPM in the adult human thyroid. CPM was not detected in NKX2-1^{+} cells or PAX8^{+} cells, although it was detected in PECAM1^{+} cells. (K) Negative expression of CPM in NKX2-1^{+} cells or PAX8^{+} cells in the fetal murine thyroid (E17.5) or adult murine thyroid (20 weeks). CPM was slightly positive in the perichondrium of the tracheal cartilage in the fetal mice. Tr: trachea.

The values are presented as the mean ± SEM. # $p < 0.05$. * $p < 0.01$. Scale bars, 100 μm.

Figure S2. Related to Figure 2.

(A) Flow cytometry of CPM^{+} and EPCAM^{+} cells on Day 0 and Day 6. The number of CPM^{+} cells was almost zero for both samples (n=3). (B) Cluster of genes decreased in the EPCAM^{+}CPM^{+} cells compared with that observed in the EPCAM^{+}CPM^{-} cells (n=3). (C) NKX2-1^{+} cells in MACS-sorted CPM^{+} and CPM^{-} cells derived from VAFECs. The proportion of NKX2-1^{+} cells was analyzed by scoring the number of NKX2-1^{+} cells relative to the total number of nuclei in an average of five randomly selected images (n=3). (D) Flow cytometry of NKX2-1^{+} cells in MACS-sorted CPM^{+} and CPM^{-} cells derived from

VAFECs on Day 14. Black line: anti-NKX2-1 antibody. Gray dotted line: isotype control (n=3). (E) Reseeded CPM$^+$ cells isolated on Day 14 and cultured in Step 5 medium in 2D for 14 days. The cells expressed NKX2-1 and SFTPB. (F) Whole-well imaging of the reseeded CPM$^+$ cells isolated on Day 23 and cultured in Step 5 medium in 2D for 14 days. The cells expressed NKX2-1 and SFTPB ubiquitously.

The values are presented as the mean ± SEM. Scale bars, 100 μm.

Figure S3, Related to Figure 3.

Normal karyotypes of the *SFTPC-GFP* reporter hPSC lines (A17-14 and B2-3).

Figure S4, Related to Figure 4.

(A) Hematoxylin-eosin staining of spheroids derived from CPM$^+$ cells and CPM$^-$ cells in VAFECs. Both cell populations formed spheroids with an intraluminal space. (B) CPM$^-$ cell-derived spheroids expressing EPCAM. (C) Expression of various markers of AECs in the CPM$^+$ cell-derived spheroids. (D) RT-qPCR comparing the 2D and 3D differentiation into AECs in the B2-3 *SFTPC-GFP* knock-in hPSC line (n=3). Each value was normalized to the level of *β-ACTIN*. The gene expression level observed in the fetal lungs was set at 1.

FHLFs: fetal human lung fibroblasts. (E) RT-qPCR comparing CPM^+ and CPM^- cell-derived 3D co-culture differentiation in H9 hESCs and 201B7 (parental), 604A1 and 585A1 hiPSCs for *NKX2-1*, *AQP5*, *FOXJ1*, *AGR2*, *KRT5*, *PAX6*, *PAX8*, *FOXN1*, *ALB* and *PDX1* (n=3). The levels of positive controls in the fetal human lungs (*NKX2-1*, *AQP5*, *FOXJ1*, *AGR2* and *KRT5*), thyroid (*PAX8*) and liver (*ALB*), postnatal thymus (*FOXN1*) and the adult brain (*PAX6*) and pancreas (*PDX1*) were set at 1.

The values are presented as the mean ± SEM. # $p < 0.05$. * $p < 0.01$. Scale bars, 100 μm.

Table S1, Related to Figure 1, 2, 3 and 4. Primers used in the present study.

Table S2, Related to Figure 1, 2, 3 and 4. Antibodies used in the present study.

SUPPLEMENTAL EXPERIMENTAL PROCEDURES

Culture of hPSCs

hESCs and hiPSCs (H9, 201B6, 201B7, 253G1, 585A1, 604A1 and 648A1) were cultured on feeder cells of mitomycin C-treated STO cells in Primate ES medium (ReproCell) supplemented with 50 U/ml of penicillin/streptomycin (Life Technologies) and 4 ng/ml recombinant human basic fibroblast growth factor (Wako). Passaging was performed using CTK dissociation solution containing 0.25% trypsin (Life Technologies), 0.1% collagenase IV (Life Technologies), 20% KSR (Life Technologies) and 1mM of $CaCl_2$ in PBS with a split ratio of 1:2 or 1:3.

2D differentiation

When the hPSCs reached 70% confluence (Day 0), the cells were incubated in 10 μM of Y-27632 (Wako) for one hour followed by deprivation of STO feeder cells with CTK dissociation solution. The cells were subsequently rinsed carefully with PBS and incubated in Accutase (Innovative Cell Technologies) for 20 minutes at 37°C. The detached hPSCs were then dissociated into single cells via pipetting, incubated on a 0.1% gelatin-coated plate for 30 minutes at 37°C and seeded on Matrigel-coated plates (BD Biosciences) at a

density of 1.1 x10^5 cells/cm^2 in the Step 1 medium containing RPMI1640 medium (Nacalai Tesque), 1x B27 supplement (Life Technologies, #17504-044) as the basal medium in addition to 50 U/ml of penicillin/streptomycin, 100 ng/ml of human activin A (R&D systems) and 1 μM of CHIR99021 (Axon Medchem), supplemented with 10 μM of Y-27632 (Day 0) and 0.25mM (Day 1) and 0.125mM (Day 2-6) of sodium butyrate (Kajiwara et al., 2012).

From Step 2 to Step 4, the basal medium consisted of DMEM/F12 plus Glutamax (Life Technologies), 1x B27 and N2 supplements (Life Technologies, #17502-048), 50 U/ml of penicillin/streptomycin, 0.05 mg/ml of L-ascorbic acid (Sigma-Aldrich), 0.4 mM of monothioglycerol (Wako), as previously reported (Green et al., 2011).

On Day 6, the medium was changed to Step 2 medium, containing the basal medium with 100 ng/ml of human recombinant noggin (R&D systems) and 10 μM of SB-431542 (R&D systems) (Green et al., 2011). On Day 10, the medium was changed to Step 3 medium, containing the basal medium with 100 ng/ml of human recombinant BMP4 (Humanzyme), 0.05-1.0 μM of all-trans retinoic acid (ATRA) (Sigma-Aldrich) and 1.5-3.5 μM of CHIR99021. The optimal concentration of ATRA/CHIR99021 was 0.5 μM/3.5 μM for H9 hESCs, 0.05 μM/2.5 μM for 201B7 hiPSCs, 0.05 μM/2.5 μM for 253G1 hiPSCs,

0.05 μM/3.5 μM for 201B6 hiPSCs, 0.5 μM/3.5 μM for 585A1 hiPSCs, 1.0 μM/2.5 μM for 604A1 hiPSCs, and 1.0 μM/2.5 μM for 648A1 hiPSCs, respectively.

On Day 14, the medium was changed to Step 4 medium in each protocol. For FGF10-based 2D differentiation (Figure 1), the medium was changed to that containing the basal medium with 100 ng/ml of human recombinant FGF10 (Wako).

On Day 21, the medium was changed to Step 5 medium consisting of Ham's F12 (Wako, #087-08335), 50 nM of dexamethasone (Sigma-Aldrich), 0.1 mM of 8-Br-cAMP (Biolog Life Science Institute, #B007), 0.1 mM of 3-Isobutyl-1-methylxanthine (IBMX) (Wako), 100 ng/ml of KGF (Wako), 0.25% of BSA (Life Technologies), 15 mM of HEPES (Sigma-Aldrich), 0.8 mM of $CaCl_2$ (Nacalai Tesque), 0.1% ITS premix (BD Biosciences), 50 U/ml of penicillin/streptomycin (Longmire, et al., 2012).

For WNT3A/FGF10/KGF-based 2D differentiation (Figure 4G and S4D), the medium was changed on Day 14 to that containing the Step 4 basal medium with 100 ng/ml of human recombinant WNT3A (R&D systems), 10 ng/ml of FGF10, and 10 ng /ml of KGF for eight days. (Green et al., 2011).

For FGF2/FGF10-based 2D differentiation (Figure 4G and S4D), the medium was changed on Day 14 to that containing the Step 4 basal medium with 500 ng/ml of basic

FGF and 100 ng/ml of FGF10 for seven days, followed by Step 5 medium for three days (Longmire et al., 2012). Each medium was replaced every two days throughout the differentiation process.

Flow cytometry

The cells were incubated in 10 μM of Y-27632 for one hour, followed by dissociation with Accutase for 20 minutes at 37°C. The detached cells were diluted in DMEM/F12 (Life Technologies) with 2% FBS and centrifuged at 800 rpm at room temperature (RT). The pellets of the cells were immersed with 1%BSA/PBS and centrifuged again. The samples were incubated in the primary antibodies for 15 minutes, washed twice with 1%BSA/PBS, and if necessary, incubated in the secondary antibodies for 15 minutes. After rinsing with 1% BSA/PBS twice, the cells were analyzed. The measurements were obtained using a BD FACSAria II flowcytometer (BD Biosciences). Isotype controls were used for gating CPM^+ or $EPCAM^+$ cells, whereas negative control cells that do not express GFP were used for gating GFP^+ cells.

Isolation of CPM^+ cells from VAFECs.

Induced VAFECs were washed twice with PBS supplemented with 0.5 mM of EDTA and dissociated with Accutase at 37°C for 20 minutes. After resuspending the detached cells in four times the volume of DMEM/F12 containing 2% FBS, the cell clumps were removed using a cell strainer with a 40-μm pore size (BD falcon).

For fluorescence-activated cell sorting (FACS), the dissociated cells were washed in 1% BSA/PBS, centrifuged at 800 rpm for five minutes, resuspended and incubated in mouse anti-human CPM antibody (0.4 μl/$1.0x10^6$ cells, diluted to 1:200 in 1% BSA/PBS) (Leica Microsystems) labeled with the Alexa 647-conjugated Fab fragment (Life Technologies) and FITC-conjugated mouse anti-human EpCAM antibody (BD Biosciences) at 4°C for 15 minutes. After rinsing with 1% BSA/PBS twice, the $EpCAM^+CPM^+$ and $EpCAM^+CPM^-$ cells were sorted using the BD FACSAria II flowcytometer (BD Biosciences).

For magnetic activated cell sorting (MACS), the dissociated cells were resuspended and incubated in mouse anti-human CPM antibody solution at 4°C for 15 minutes. After washing twice with 0.5% BSA/PBS with 2mM EDTA, the labeled cells were incubated in anti-mouse IgG1 microbeads solution (20 μl/$1x10^7$ cells, diluted to 1:4 in 0.5% BSA/PBS with 2mM EDTA) at 4°C for 15 minutes. After washing twice with the same

buffer, the CPM^+ cells were separated using a magnetic stainless column (Miltenyi Biotec). To improve purity, separation was performed twice, and the number of harvested cells was counted. 10 μM of Y-27632 was supplemented for all processes of FACS and MACS until sorting of the CPM^+ and CPM^- cells was completed.

3D differentiation

A total of $2.0x10^4$ CPM^+ cells isolated from induced VAFECs on Day 14 were mixed with $1.0x10^6$ of fetal human lung fibroblasts (17.5 weeks of gestation, DV Biologics, # PP002-F-1349, Lot.121109VA) in 1:1 Matrigel/Step 5 medium, and a total volume of 400 μl was seeded on a 12-well cell culture insert (BD Biosciences, #353180) with 10 μM of Y-27632 and 1 ml of Step 5 medium placed in the lower chamber. The cells were maintained by changing the medium in the lower chamber every other day for 10 days. For the FACS analyses, Matrigel blocks containing differentiated cells and fibroblasts were minced into small pieces and incubated in 0.1% Trypsin/0.5 mM EDTA at 37°C for 15 minutes with occasional pipetting.

Quantitative RT-PCR

Total RNA was isolated using the PureLink RNA Mini Kit (Life Technologies) according to the manufacturer's manual. First-strand cDNA was synthesized from 80 ng of total RNA using the SuperScript III First-Strand Synthesis System (Life Technologies). The cDNA samples were amplified using Power SYBR Green PCR Master Mix with ABI7300 Real-Time PCR System (Life Technologies). All reactions were started at a cycle of 95°C for 10 minutes, followed by 45 cycles of 95°C for 15 seconds, 62°C for 20 seconds and 72°C for 30 seconds. The PCR reactions were performed in triplicate for each sample. The level of expression of each gene was calibrated to that of the housekeeping gene, *β-ACTIN*, and compared to the level of the expression of each gene in the fetal human lungs (17, 18, and 22 weeks gestation, Agilent Technologies, #540177, Lot.0006055802). All primer sets are shown in Table S1.

Immunofluorescence staining

The tissue cryosections and cells were fixed with 4% paraformaldehyde /PBS (Nacalai Tesque) for 15 minutes at RT. After washing three times with PBS, the cells were immersed in 0.2% Triton X-100 /PBS for 15 minutes at RT, followed by incubation with the blocking solution consisting of 5% normal donkey serum (Millipore), 1% BSA

(Sigma-Aldrich), and PBS (Nacalai Tesque) for 30 minutes at RT. The cells were incubated in the primary antibody solution for 30 minutes at RT, followed by washing three times with 1% BSA/PBS, after which they were incubated in the secondary solution for 30 minutes at RT and washed three times with PBS. Regarding the results of staining for GFP and SFTPC, we concerned about following things. First, we avoided freezing the spheroids embedded in the OCT compounds (Sakura Finetek) before incubation with the primary anti-GFP (Aves Labs) and anti-proSPC antibodies (EMD-Millipore) in order to detect the gene expression in the spheroids (Figure 4D), due to the potential loss of antigenicity caused by freezing. Instead, whole-mount fixation and staining were performed before freezing and sectioning. Second, we also avoided use of anti-proSPC antibody (EMD-Millipore) to stain the cells in the two-dimensional culture, as nonspecific staining inconsistent with the results of RT-qPCR of *SFTPC* was observed. Indeed, nonspecific reactions of other commercial anti-proSPC antibody to cells in two-dimensional culture have recently been reported (Schmeckebier et al., 2013). Therefore, anti-SFTPC antibody (Santa Cruz Biotechnology) were used for the two-dimensional culture and staining of other tissue/spheroid sections. Corresponding gene expression analyses of *SFTPC* were performed using RT-qPCR. All primary and secondary antibodies used in the present study

were diluted in blocking solution as indicated in Table S2. Nuclei were counterstained with Hoechst-33342.

Generation of SFTPC-reporter knock-in hPSCs

The human BAC clones of RP11-102O8 and CTD-2530N21 both of which contains all of the exons of the *SFTPC* gene and extends from 138.1 kb upstream to 39.3 kb downstream of the gene locus for the former clone and 92.9 kb upstream to 123.8 kb downstream for the latter clone, were purchased from Life Technologies. Gene targeting was performed as previously described (Mae, et al., 2013). In order to construct the knock-in vector, we designed two primers with 60 bp sequences of the homologous recombination regions of the *SFTPC* gene with the 5' or 3' end of the *EGFP-pA-PNL* sequence (kindly provided by Dr. Kazutoshi Takahashi), and performed genomic PCR using KOD Plus Neo polymerase (Toyobo) according to the manufacturer's protocol. The primers used for genomic PCR were as follows: hSFTPC-EGFP-F, ATATAAGACCCTGGTCACACCTGGGAGAGGAGGAGAGGAGAGCATAGCACCTG CAGCAAGATGGTGAGCAAGGGCGAGGA; hSFTPC-PNL-R, CCCATCACACACATG TGCGCGCACACATACATACACACACGCAACCACACTCACCGGCGTCGACGGC

GAGCTCAGACG. The targeting vector, the *EGFP-pA-PNL* cassette containing 5' and 3' homology arms, was then electroporated into DH10B containing BAC RP11-102O8 and CTD-2530N21, respectively, and activated recombinases using the Red/ET-mediated recombination technique (Gene Bridges). The transformed bacteria were plated on LB plates with appropriate antibiotics and incubated overnight at 37°C. Selected clones were subjected to PCR to confirm whether the *EGFP-pA-PNL* cassette was integrated into the *SFTPC* endogenous locus via successful homologous recombination. The RP11-102O8 derived-targeting vector was named A17 and the CTD-2530N21 derived-targeting vector was named B2. The human *SFTPC-EGFP-pA-PNL* BAC vector was electroporated into hiPSCs (201B7), and the electroporated cells were plated on feeder layers of mitomycin C-treated STO cells. G418 antibiotic selection was applied two days after electroporation until each colony was picked up manually. In order to select positive clones in which homologous recombination occurred, real-time PCR reactions were carried out with 100 ng of genomic DNA using the predesigned TaqMan copy number assay (Assay ID: SFTPCdel_CCCSUW0). Among 55 G418-resistant clones, 12 clones were selected with a heterozygous deletion of the endogenous *SFTPC*-coding region replaced by the *GFP-pgk-Neo* cassette (Figure 3B). Because many candidate clones were obtained, two

clones, B2-3 and A17-14, were chosen for use in the subsequent studies. The hiPSCs with a targeted *SFTPC* allele were treated with 10 μM of Y27632 overnight and trypsinized. Cells resuspended in PBS were electroporated with 30 μg of pCXW-Cre-Puro (kindly provided by Dr. Keisuke Okita) and plated on feeder layers of mitomycin C-treated puromycin-resistant STO cells (kindly provided by Dr. Kazutoshi Takahashi). Antibiotic selection with 1 μg/ml of puromycin was applied two days after electroporation for two days. Conventional PCR was performed to confirm the loss of the *PGK-Neo* cassette. G-band analyses were carried out at Nihon Gene Research Laboratories, Japan. Genomic DNA was analyzed using CytoScan HD arrays (Affymetrix) according to the manufacturer's protocol. The genomic copy numbers were calculated using the CNAG3.5.1 software program (Nannya et al., 2005; Yamamoto et al., 2007). Genetic lesions, including copy number gains and losses, were detected using a hidden Markov model (HMM)-based algorithm implemented in the CNAG3.5.1 software program (http://www.genome.umin.jp/CNAG_DLpage/CNAG_top.html). RT-PCR of *GFP* and *SFTPC* were performed as follows: initial denaturation was 95°C for 10 minutes, followed by 25 cycles (*β-ACTIN*) or 30 cycles (*GFP* or *SFTPC*) of 95°C for 15 seconds, 62°C for 20 seconds and 72°C for 30 seconds.

Gene expression studies using microarrays

Biotinylated cRNA was synthesized using the GeneChip 3'IVT Express Kit (Affymetrix) from 250 ng of total RNA according to the manufacturer's instructions. A total of 10 ug of cRNA was hybridized for 16 hours at 45°C on the GeneChip Human Genome U133 Plus 2.0 Array, followed by washing and staining in the Affymetrix Fluidics Station 450. The GeneChips were scanned using the GeneChip Scanner 3000 7G, and the resulting images were quantified and normalized according to the MAS5.0 algorithm. The trimmed mean target intensity of each chip was arbitrarily set to 500. The data were processed using Gene Spring GX software program.

Electron microscopy

3D matrices involving differentiated cells were cut into small pieces and incubated in fixative containing 2.5% glutaraldehyde, 2% paraformaldehyde, 2% osmium tetroxide, 0.1% picric acid, 4% sucrose, and 0.1M phosphate buffer (pH 7.4) at 4°C for two hours, followed by incubation in 1% uranyl acetate en bloc at RT for one hour (Osanai et al., 2010). The samples were washed in an ascending concentration of ethanol and propylene

oxide and embedded in Epon 812. Thin sections were doubly stained with uranyl acetate and lead citrate and examined under a Hitachi H-7650 transmission electron microscope.

Human and murine tissues and total RNA samples

The following human specimens were obtained from DV Biologics (Canada), Agilent Technologies (United States), and Biochain (United States); total RNA of fetal lung (17, 18, and 22 weeks gestation, Agilent Technologies, #540177, Lot.0006055802), that of adult lung (40 years of age, Agilent Technologies, #540019, Lot.0006118369), that of fetal thyroid (36 weeks gestation, Biochain, #R1244265-10, Lot.A811069), that of adult thyroid (38, 60, and 68 years of age, Life technologies, #AM6000, Lot.1102082), that of fetal throat (36 weeks gestation, Biochain, # R1244263-10, Lot.B212133), that of fetal liver (20 weeks gestation, Biochain, # R1244149-50, Lot.A601605), that of postnatal thymus (6, 6, and 10 months of age, Life technologies, #AM6000, Lot.1102082), that of adult brain (59, 78, and 85 years of age, Life technologies, #AM6000, Lot.1102082), that of adult pancreas (83 years of age, Life technologies, #AM7954, Lot.0910004), frozen tissue of fetal lung (18.5 weeks gestation, DV Biologics, PP001-FS, Lot.102508RH), that of adult thyroid (25 years of age, Biochain, #T1234265-RT1, Lot.A709031), and fetal lung fibroblasts (17.5 weeks

gestation, DV Biologics, # PP002-F-1349, Lot.121109VA). All murine specimen were derived from C58BL/6 strain.

SUPPLEMENTAL REFERENCES

Nannya Y, Sanada M, Nakazaki K, Hosoya N, Wang L, Hangaishi A, Kurokawa M, Chiba S, Bailey DK, Kennedy GC, Ogawa S. (2005). A robust algorithm for copy number detection using highdensity oligonucleotide single nucleotide polymorphism genotyping arrays. Cancer Res. *65*, 6071–6079.

Osanai K, Higuchi J, Oikawa R, Kobayashi M, Tsuchihara K, Iguchi M, Huang J, Voelker DR, Toga H. (2010). Altered lung surfactant system in a Rab38-deficient rat model of Hermansky-Pudlak syndrome. Am. J. Physiol. Lung Cell Mol. Physiol. *298*, L243-251.

Schmeckebier S, Mauritz C, Katsirntaki K, Sgodda M, Puppe V, Duerr J, Schubert SC, Schmiedl A, Lin Q, Paleček J, Draeger G, Ochs M, Zenke M, Cantz T, Mall MA, Martin U. (2013). Keratinocyte growth factor and dexamethasone plus elevated cAMP levels synergistically support pluripotent stem cell differentiation into alveolar epithelial type II cells. Tissue Eng. Part A. *19*, 938-951.

Yamamoto G, Nannya Y, Kato M, Sanada M, Levine RL, Kawamata N, Hangaishi A,

Kurokawa M, Chiba S, Gilliland DG, Koeffler HP, Ogawa S. (2007). Highly sensitive method for genomewide detection of allelic composition in nonpaired, primary tumor specimens by use of affymetrix single-nucleotide- polymorphism genotyping microarrays. Am. J. Hum. Genet. *81*, 114–126.
